# HOXA10 drives immune evasion in early lung adenocarcinoma by recruiting immunosuppressive macrophages via NF-κB/CCL2 signaling

**DOI:** 10.1186/s13046-026-03679-6

**Published:** 2026-02-25

**Authors:** Jiakang Ma, Xingyu Xu, Jialiang Zhu, Jie Wang, Li Wang, Yun Liu, Wenqing Qiu

**Affiliations:** 1 MOE Key Laboratory of Metabolism and Molecular Medicine, Department of Biochemistry and Molecular Biology, School of Basic Medical Sciences, Fudan University, Shanghai, China; 2 Department of Thoracic Surgery and State Key Laboratory of Genetic Engineering, Fudan University Shanghai Cancer Center, Shanghai, China; 3 R&D Department, Suzhou Jiyan Biopharmaceutical Technology, Taicang Biomedical Industrial Park, Suzhou, China; 4 MOE Medical Basic Research Innovation Center for Gut Microbiota and Chronic Diseases, Wuxi School of Medicine, Jiangnan University, Wuxi, China; 5 Department of General Surgery, Central Laboratory, Shanghai Xuhui Central Hospital, Zhongshan-Xuhui Hospital, Fudan University, Shanghai, China

**Keywords:** Early-stage LUAD, HOXA10, CCL2, Immune evasion, NF-κB

## Abstract

**Background:**

Immune evasion is a critical determinant of cancer progression, yet mechanisms underlying early-stage acquisition of these capabilities remain poorly understood. This study builds an early lung adenocarcinoma mouse model to explore the key regulator of tumor immune evasion process in the very early stage of human lung adenocarcinoma.

**Methods:**

We introduced Kras^G12D/+^ and Trp53^−/−^ oncogenic mutations into murine lung organoids to model newly transformed lung adenocarcinoma cells. Sequential orthotopic injections subjected these cells to iterative immune selection pressure, facilitating the progressive acquisition of immune evasion capabilities. Transcriptomic analyses revealed progressive upregulation of HOXA10, a homeobox transcription factor, throughout the in vivo immune selection process. The tumor microenvironment was evaluated through single-cell RNA sequencing, flow cytometry, and immunohistochemical analysis. Chromatin immunoprecipitation (ChIP) assays, dual-luciferase reporter assays, enzyme-linked immunosorbent assays (ELISA), migration assays, and Western blot analysis were performed to identify downstream targets and elucidate the mechanism by which HOXA10 mediates immune evasion. Immune checkpoint blockade (ICB) treatment was administered to investigate the role of HOXA10 in cancer immunotherapy.

**Results:**

Our study demonstrated that HOXA10 is upregulated in our lung adenocarcinoma mouse model and in early-stage clinical lung adenocarcinoma specimens. Patients with higher HOXA10 expression exhibited significantly poorer survival. Hoxa10 knockdown substantially inhibited tumor growth, increased cytotoxic CD8^+^ T cell infiltration, reduced tumor-infiltrating barrier (TIB) density at tumor margins, and decreased immunosuppressive macrophage populations while enhancing macrophage-CD8^+^ T cell interactions. Mechanistically, Hoxa10 directly binds to the promoter region of the *Ikbkb* gene, which encodes IKKβ protein, thereby promoting its expression. Upregulated IKKβ enhanced nuclear translocation of p65, consequently augmenting downstream Ccl2 expression. Furthermore, we demonstrated that Hoxa10 suppression significantly enhanced immunotherapeutic efficacy.

**Conclusion:**

During early lung adenocarcinoma development, tumor cells acquire immune evasion capabilities through HOXA10 upregulation, which remodels the intra-tumoral immune microenvironment and promotes adenocarcinoma initiation and progression. This mechanism represents a novel therapeutic target for early-stage lung cancer intervention and highlights the importance of immune tolerance in early-stage cancer.

**Supplementary Information:**

The online version contains supplementary material available at 10.1186/s13046-026-03679-6.

## Background

Lung adenocarcinoma (LUAD) is the most prevalent subtype of non-small cell lung cancer (NSCLC), accounting for approximately 80% of all NSCLC cases. Although immune checkpoint blockade (ICB) has significantly improved overall survival in a subset of LUAD patients, nearly 70% of patients develop resistance through mechanisms that remain largely undefined [1]. Identifying the mechanisms that orchestrate immune dysfunction within the tumor microenvironment is vital for improving clinical immunotherapy efficacy. However, current research has primarily focused on advanced-stage LUAD, which have exhibited well-established immunosuppressive ecosystems [2, 3]. In contrast, the mechanisms responsible for initiating this immunosuppressive landscape during the earliest phases of tumorigenesis are still largely unknown, representing a significant gap in our knowledge.

The progression from pre-neoplastic lesions to invasive lung adenocarcinoma is accompanied by dynamic remodeling of the immune landscape. Signatures of immune suppression are detectable even at the pre-invasive stage [4]. Importantly, recent multimodal spatial-omics studies of lung precursor lesions have revealed that alveolar progenitor cells co-evolve with pro-inflammatory niches enriched in specific macrophage subsets from the earliest phases of disease development, underscoring the important role of epithelial-macrophage interactions in tumor initiation [5]. Tumor-associated macrophages (TAMs), particularly those with an M2-polarized phenotype, serve as pivotal architects of this immunosuppressive niche. They secrete cytokines such as IL-10, TGF-β, and MMP9, which trigger the epithelial-mesenchymal transition (EMT), remodeling of the extracellular matrix (ECM), and recruitment of other immunosuppressive cells [6]. Their abundance in LUAD is frequently associated with poor prognosis and resistance to immunotherapy [7]. Despite their recognized involvement in both early lesions and advanced disease, the key molecular drivers that orchestrate the recruitment and polarization of pro-tumorigenic macrophages during the initial immune-editing phase remain poorly defined.

The homeobox transcription factor HOXA10, crucial in embryonic patterning, has emerged as an oncogenic driver in several malignancies [8, 9]. In LUAD, HOXA10 is consistently upregulated and promotes tumor proliferation, invasion, and metastasis [10]. Notably, in gastric cancer, HOXA10 can transcriptionally activate and increase the secretion of TGFB2, a potent immunomodulatory cytokine, thereby activating the downstream TGFβ/Smad signaling pathway [8]. However, its specific role in shaping the immunosuppressive landscape of LUAD, particularly its potential connection to recruiting and instructing pro-tumorigenic macrophages during early immune evasion, remains entirely unexplored.

To address this challenge, we developed a novel murine LUAD model to capture tumor evolutionary dynamics and tumor-immune interactions across multiple early timepoints. We first established mouse lung organoids harboring *Kras* activation and *Trp53* deficiency-recapitulating the foundational genomic drivers of human LUAD initiation [11]. By subjecting these oncogenically initiated cells to iterative rounds of in vivo immune selection through orthotopic transplantation, we successfully modeled the evolutionary trajectory toward immune evasion. Longitudinal transcriptional profiling identified that the transcription factor HOXA10 as being progressively and markedly upregulated, linking its expression to the acquisition of immune-evasion capabilities. Mechanistically, HOXA10 orchestrates a potent immunosuppressive program by transactivating *CCL2*, which recruits immunosuppressive macrophages to the tumor microenvironment. This reshaped TME ultimately impairs CD8⁺ T cell infiltration and cytolytic activity, equipping early LUAD cells with the necessary machinery to bypass immunosurveillance and drive malignant progression.

## Materials and methods

### Animal experiments

Kras^*LSL−G12D/*+^/Trp53^*fl/fl*^ mice were utilized for lung organoid culture. Female C57BL/6 J mice aged 6–8 weeks were used for the development of subcutaneous and orthotopic tumor models.

For the “in vivo cycle” system, the early tumor cells were exposed to iterative rounds of immune selection within the lung microenvironment. To strike a balance between minimizing rapid tumor outgrowth and ensuring adequate recovery of EGFP^+^ cells following short-term exposure, an optimized inoculum of 5 × 10^5^ cells per injection was used. AP-T1 or AHS group cells (5 × 10^5^ cells) were mixed with Matrigel (Corning, #356,230) at a 1:1 ratio and orthotopically injected into the left lung lobe of C57BL/6 J mice. After 48 h, lung tissues were harvested, enzymatically digested and cultured in complete DMEM/F12 medium. After 2–3 passages, EGFP-positive cells were enriched and reinjected orthotopically.

For the subcutaneous tumor model, 2 × 10^6^ tumor cells were subcutaneously injected into the left groin. At day 16, tumors were harvested, and tumor weight and volume were measured. Tumor volume was calculated using the formula: volume = length × (width)^2^/2. Subsequently, tumors were collected for hematoxylin and eosin staining and histological analysis.

For the mouse orthotopic lung tumor model, 5 × 10^6^ tumor cells were mixed with Matrigel at a 1:1 ratio and orthotopically injected into the left lung lobe with single volume of 50 μl [12, 13]. After 6 weeks, mice were euthanized, and lungs were harvested for subsequent tumor growth analyses and immune profiling.

For immune checkpoint blockade therapy with anti-PD-L1 in the mouse tumor model, subcutaneous tumor models were established as described above. On day 7, tumor volumes were measured for the first time. Mice injected with the same tumor cell type (shCTL or shHoxa10 cells) were randomly divided into two groups to minimize the impact of batch effects on the experimental outcomes (for isotype or anti-PD-L1 mAb treatment). The indicated groups were subsequently treated intraperitoneally with 100 μg anti-PD-L1 mAbs (BioXCell #BE0101) or IgG control on day 7, 10, and 13. On day 19, tumors were harvested with tumor weight and volume measured. Subsequently, tumors were collected for hematoxylin and eosin staining and histological analysis. To adhere to ethical guidelines, a maximum allowable tumor volume of 2000 mm^3^was set. Daily monitoring was performed, and mice were euthanized upon reaching this endpoint.

### Organoid generation

Lung tissue of *Kras*^*LSL−G12D/*+^*/Trp53*^*fl/fl*^ C57BL/6 J mouse was dissected, minced, and dissociated using 1.5 ml dissociation medium (1 × collagenase/hyaluronidase, Stem Cell Technologies, #07912). The dissociated tissue was further digested with TrypLE (Gibco, #12,605–028). After centrifugation, cells were suspended by mouse lung organoid medium and mixed with equal volume of Matrigel. 50 μl of the mixture containing 2 × 10^4^ to 4 × 10^4^ cells per droplet were seeded into 24-well plate according to the manufacturer’s instructions (Suzhou Jiyan Biotech. Co. Ltd., China, MOCKs004-B). Lung organoids were cultured for 7 days after initial isolation and passaged every 5 days. Lentiviral infection with Cre-EGFP-expressing vectors was performed after stable organoid formation on day 28. After 2–3 passages (about 8–10 days due to the selective growth advantage to the transformed organoids), the proportion of EGFP-positive cells within the lung organoids gradually increased to over 70%. The genotype of these EGFP-positive organoids was confirmed. Subsequently, EGFP-positive organoids were cultured without Matrigel in mixed cell culture medium (1:1 ratio of mouse lung organoid medium and DMEM/F12 supplemented with 1% penicillin–streptomycin and 10% FBS) for 2 weeks to promote cell attachment.

### Cell culture

#### AP group cells

This group serves as the in vitro control, consisting of cells maintained in parallel culture without in vivo exposure. Starting from the initial 2D-cultured EGFP-positive organoid cells (designated AP T1 at day 0), cells were continuously passaged in vitro and harvested at time points matched to the corresponding AHS group.

#### AHS group cells

This group comprises cells subjected to sequential rounds of in vivo immune selection. Specifically, AP T1 cells underwent the first orthotopic injection and recovery cycle to generate AHS T2. AHS T2 cells were then subjected to a second in vivo cycle to yield AHS T3, and the process was repeated to produce AHS T4. Cells derived through this iterative in vivo selection are collectively referred to as the AHS group.

All AHS and AP group cells, including Hoxa10 knockdown and overexpression cells, were cultured in complete DMEM/F12 medium (Meilunbio). This complete medium consisted of DMEM/F12 supplemented with 1% penicillin/streptomycin (Gibco) and 10% fetal bovine serum (FBS, Gibco). Hoxa10 overexpression cells were treated with JSH-23 (15 μM) for 24 h to inhibit p65 translocation.

### Lentiviral production and infection

AHS-T4 was selected as the endpoint of our in vivo cycle process and demonstrated the most potent tumorigenic and immune-evasive properties among all tested groups. Therefore, AHS-T4 cells were used as the parental line to generate the stable knockdown (shHoxa10), overexpression (Hoxa10-OE), and control (shCTL) lines for subsequent mechanistic studies.

The coding sequences of Hoxa10 was amplified from cDNA of LLC1 cell line and inserted into the pLVX-EF1a vector. For shRNA constructs, the pLKO.1-puro vector was used. Lentiviral particles expressing shRNAs were produced using a third-generation packaging system in HEK293T cells (ATCC, CRL-11268). The target plasmids were transfected into HEK293T cells together with envelope plasmid pVSVG, packaging plasmids pRSV-Rev, and pMDLg/pRRE using PEI to produce lentivirus. Viral supernatant was harvested every 48 h over a period of 4 days, then filtered and either stored at –80 °C or used directly for infection. Target cells at 70–80% confluence were infected with the viral supernatant supplemented with 8 μg/ml polybrene (Yeasen). Infected cells were selected using puromycin (Beyotime) at 1 μg/ml for AHS-T4 cells, which was added 48 h post-infection. After 7 days of selection, surviving cells were passaged.

### Generation of bone marrow-derived macrophages

Bone marrow-derived macrophages (BMDMs) were prepared as previously described [14–16]. Briefly, bone marrow was isolated from mouse femurs and tibias. The isolated cells were stimulated with CSF-1 (10 ng/mL) and cultured for 7 days. After stimulation, F4/80 expression on cell surface were tested by flow cytometry to ensure cell differentiation.

### Cell migration

BMDMs were resuspended in serum-free DMEM medium to achieve a concentration of 2 × 10^5^ cells/ml. For each assay, 0.2 ml of the cell suspension was seeded into 8-μm Transwell inserts (Corning). The lower chambers were seeded with 5 × 10^5^ stable tumor cells and filled with 0.5 ml of RPMI-1640 supplemented with 10% FBS. After 48 h of incubation, cells remaining on the upper surface of the membranes were gently removed. The membranes were then fixed and stained with crystal violet (Sangon Biotech) for 2 h. Cells that had migrated through the membrane were quantified by counting in five different microscopic fields per membrane at 20 × magnification. Data were collected from five replicate inserts per condition. Statistical analyses were performed using a two-tailed t-test.

### Cell coculture

For cell coculture experiments, we used a Transwell system which was an indirect coculture system. 5 × 10^5^ BMDM cells were seeded into the lower chamber of Transwell with 0.2 ml complete DMEM medium, and 5 × 10^5^ stable tumor cells were seeded into 8-μm Transwell inserts. After 48 h of coculture, BMDM cells were harvested and processed into single-cell suspensions for flow cytometry analysis.

### Quantitative RT-PCR

RNA samples were extracted from cultured cell lines using the RNeasy Kits (QIAGEN, #74,104) according to the manufacturer's instructions. The isolated RNA was reverse transcribed into cDNA using PrimeScript RT Master Mix (Takara: RR036B) following the manufacturer's protocol. Quantitative RT-PCR analysis was performed using ChamQ Universal SYBR qPCR Master Mix (Vazyme: Q711-02) according to the manufacturer's instructions. RT-PCR was conducted on a LightCycler 480 Instrument II (Roche). The sequences of primers used in this study are listed in Table S4.

### Western blot and antibodies

After two washes with PBS, cells were lysed using RIPA buffer (Cell Signaling Technology, 9806S) and incubated at 4 °C for 10 min, followed by centrifugation at 12,000 × g for 10 min. The supernatant was collected and boiled for 5 min. Protein lysates were separated on 10% SDS-PAGE gels and subsequently transferred to PVDF membranes (Millipore, #IPVH00010). The membranes were incubated with primary antibodies (listed in Supplementary Methods) overnight at 4 °C. After washing, the membranes were probed with HRP-conjugated anti-rabbit IgG (1:5,000, CST, 7074S) or anti-mouse IgG (1:5,000, CST, 7076S) for 1 h at room temperature. Signal detection was performed using the Advansta WesternBright™ Sirius ECL detection kit.

### Luciferase reporter assay

For the luciferase activity assay, AHS-T4 cells were seeded in 96-well plates at a density of 5 × 10^3^ cells per well in triplicate and cultured for 24 h. Transfection was carried out using Lipofectamine 3000 (Invitrogen) with a dosage of 0.2 μl per well according to the manufacturer’s protocol. Each well received a transfection mixture containing 100 ng of PGL4.10 luciferase reporter plasmid, 100 ng of HOXA10 expression plasmid, and 2 ng of PGL4.75 (Renilla luciferase) plasmid [17]. After 48 h, luciferase and Renilla activities were measured using the dual-luciferase reporter assay kit (Yeasen, #11,405) as directed by the manufacturer.

### Immunohistochemistry (IHC) and Immunofluorescence (IF)

Paraffin-embedded tissue sections were deparaffinized in xylene and rehydrated through a graded ethanol series (100%, 95%, 75%) followed by a rinse with Tris-buffered saline (TBS). Antigen retrieval was performed using EDTA buffer (pH 9.0) in a pressure cooker. After cooling, sections were treated with 3% hydrogen peroxide (Sinopharm) and blocked with 10% goat serum (Boster: AR1009). Primary antibodies used here were listed in Supplementary methods. Appropriate secondary antibodies (anti-rabbit, anti-goat, anti-rat, or anti-mouse; ab205718, ab6789, ab97110, ab97057) were applied and incubated at 37 °C for 45 min. DAB substrate (Abcam, ab64238) was added, and the reaction was terminated with water. The sections were counterstained with hematoxylin (Baso, BA4041), dehydrated, mounted, and air-dried or dried at 37 °C before examination under a light microscope. For immunofluorescence, cells were incubated with an anti-P65 primary antibody (1:100, Proteintech, #10,745–1-AP) overnight at 4 °C, followed by incubation with a Cy3-conjugated secondary antibody (1:500, Abcam, ab6939) at room temperature for 1 h. Nuclei were counterstained with DAPI for 10 min, and slides were mounted with ProLong Gold antifade mounting medium (Life Technologies, P36930). Images were captured using a Nikon Eclipse 90i upright microscope.

### Chromatin Immunoprecipitation (ChIP)

Cells were cross-linked with 1% formaldehyde at room temperature for 10 min, followed by addition of 125 mM glycine to quench the reaction. Cell pellets were harvested and resuspended in Lysis buffer (10 mM KCl, 20 mM Tris–HCl pH 8.0, 10% glycerol, 2 mM DTT), then gently rotated at 4 °C for 50 min to isolate nuclei. Nuclei were washed twice with cold PBS and resuspended in SDS lysis buffer (50 mM Tris–HCl pH 8.1, 10 mM EDTA, 0.5% SDS, 1 × protease inhibitor cocktail). Chromatin was sheared to approximately 300 bp fragments using an M220 Focused-ultrasonicator (Covaris). For immunoprecipitation, 70 μl of Dynabeads Protein A/G slurry (Thermo Fisher Scientific) was prepared by washing twice with blocking buffer (0.5% BSA in IP buffer) and added to each sample, followed by incubation with 6 μg of either anti-HOXA10 antibody or control IgG at 4 °C for 12 h. The supernatant was then removed. For chromatin binding, 250 μg of soluble chromatin in IP buffer (20 mM Tris–HCl pH 8.0, 2 mM EDTA, 150 mM NaCl, 1% Triton X-100, and protease inhibitor cocktail) was added to the bead-antibody complexes and incubated for 2 h. The supernatant was removed and the bead-DNA–protein complexes were washed five times with RIPA washing buffer (50 mM HEPES pH 7.6, 1 mM EDTA, 0.7% sodium deoxycholate, 1% NP-40, 0.5 M LiCl). Subsequently, Samples were first incubated overnight at 65 °C in a ChIP Elution Buffer (50 mM Tris–HCl, pH 8.1; 10 mM EDTA; 1% SDS) to reverse crosslinks. The eluted supernatant was then treated with 2 µl of RNase A and incubated at 37 °C for 30 min, followed by addition of 2 µl of Proteinase K and further incubation at 55 °C for 1 h. The immunoprecipitated DNA was purified using Mini-Elute PCR purification kits (Qiagen) and analyzed by quantitative real-time PCR.

### Flow cytometry analysis

Fresh tumor tissues were excised and enzymatically digested with collagenase IV & I (Gibco) and DNase I (Roche) for 40 min, then filtered through 70-μm cell strainers to obtain single-cell suspensions. After blocking non-specific antibody binding with CD16/CD32 antibody (CST, #88,280), surface staining was performed with relative antibodies listed in Supplementary methods. After fixation and permeabilization using a Fixation/Permeabilization kit (Invitrogen, GAS003), intracellular IFN-γ was stained with anti-IFN-γ antibody (BD, #562,020). Stained cells were analyzed using a Beckman CytoFLEX S flow cytometer. Data were analyzed using FlowJo 10.8.1 software.

### ELISA

Supernatant during stable cell line coculture with BMDM was collected. Soluable level of CCL2 in the supernatant was tested by Mouse CCL2 ELISA kit (BYabscience, #BY-EM222324) according to manufacturer`s instruction.

### Human samples

All LUAD patient samples with detailed clinical characteristics were provided in Supplementary Table S3. Tumor and adjacent non-tumor tissues were obtained during surgical procedures at the Fudan University Shanghai Cancer Center, with written informed consent compliance from all participants.

### Whole Exomes Sequencing (WES)

Whole exome sequencing (WES) was performed on the seven cell lines derived from our mouse model (AP T1 to AP T4 and AHS T2 to AHS T4). Raw sequencing data were subjected to quality control using fastp (v0.21.0) [18] with default parameters to remove adapter-containing reads, reads with poly-N stretches, and low-quality reads. The resulting paired-end clean reads were aligned to the Mus musculus reference genome (GRCm39) using BWA-MEM (v0.7.17) [19]. The aligned BAM files were then sorted and indexed using Samtools (v1.18) [20]. PCR duplicates were identified and removed with GATK MarkDuplicates (v4.1.0) [21] under default settings. The final deduplicated BAM files were used for downstream analyses. More analysis details were listed in Supplementary methods.

### RNA-seq processing

Raw sequencing data were subjected to quality control using fastp (v0.21.0) with default parameters to remove adapter-containing reads, reads with poly-N stretches, and low-quality reads. The resulting paired-end clean reads were aligned to the Mus musculus reference genome (GRCm39) using STAR (v2.7.11b) [22]. The aligned BAM files were sorted and indexed with Samtools (v1.18). PCR duplicates were identified and removed using GATK MarkDuplicates (v4.1.0). The final deduplicated BAM files were used for downstream analyses. Gene-level read counts were quantified using featureCounts (v2.1.1) [23]. More detailed methods were listed in Supplementary methods.

### scRNA-seq library preparation and data processing

Detailed methods for library preparation were listed in Supplementary methods. Read counts from scRNA-seq were obtained using the CellRanger toolkit (version 6.1.2) [24]. The mm10 genome was used as the reference for read alignment. Combined read counts were analyzed using Seurat (version 5.3.0) [25]. Cells were filtered based on three quality control metrics: (1) total UMI count between 1,200 and 40,000; (2) number of detected genes between 600 and 6,000; (3) percentage of mitochondrial gene counts less than 15%. Additionally, genes detected in fewer than 3 cells and cells detected with fewer than 200 genes were filtered out. DoubletFinder [26] was applied to each sequencing library to remove potential doublets, with the expected doublet rate set to 0.08. Batch effects were removed using Harmony [27]. Major cell types were identified based on canonical marker genes including *Cd3d, Cd3e, Nkg7, Cd79a, Cd19, Mzb1, Cd68, Cd14, Kit, and Tpsab1* [28]. Cell subtypes were then annotated as indicated in Supplementary Fig. 4. Pseudotime trajectory analysis was performed using Monocle2 (v2.22.0) [29]. Differentially expressed genes were selected using the dpFeature method with a significance threshold of q-value < 0.01. Dimensionality reduction and trajectory inference were conducted using the reduceDimension function with the DDRTree algorithm. All functions were run with default parameters unless otherwise specified. Cell ordering along the inferred trajectories was performed using the orderCells function. For trajectory initialization, the root state was defined as the Monocle state enriched for CD8T_Tn_Tcf7 cells in the CD8⁺ T cell trajectory, respectively, representing less differentiated and naïve-like T cell states.

### Statistics

All quantitative data are presented as mean ± SD. Statistical analyses were performed using GraphPad Prism 10 (GraphPad Software) or related R packages. Data in bar graphs represent fold change or percentage relative to control with SD from 3 independent experiments. Student's t-test was used to compare two groups of independent samples, and multiple comparisons were corrected using Bonferroni's method. The Mantel-Cox test was used to evaluate statistical differences between groups in survival studies. A *P* value of less than 0.05 was considered statistically significant. Significance levels were indicated as follows: *P < 0.05, **P < 0.01, ***P < 0.001, and ****P < 0.0001. R (version 4.2.0) was used for statistical testing.

## Results

### Acquisition of immune evasion potential in early LUAD throughin vivoselection

**Fig. 1:**
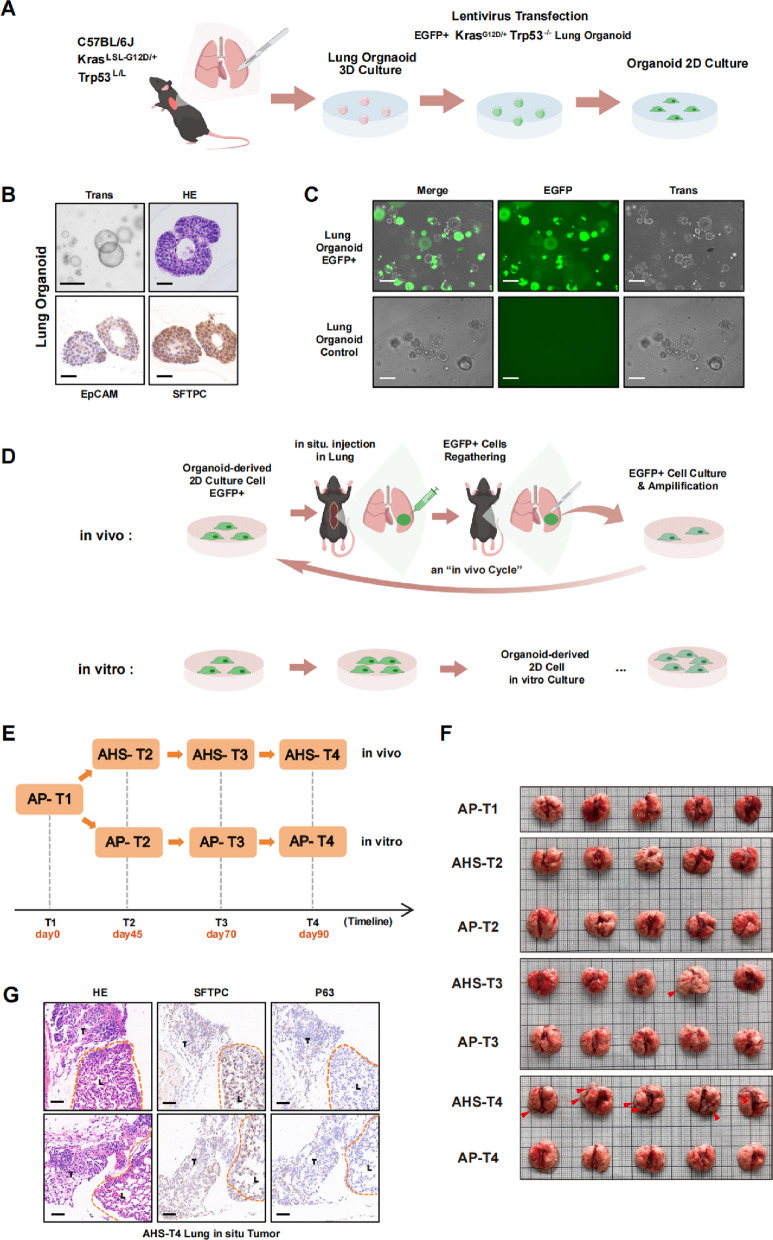
An Orthotopic Immune Selection Model Reveals the Acquisition of Immune Evasion in Early Lung Adenocarcinoma. **A** Schematic of the strategy for generating EGFP + *Kras*^G12D/+^/*Trp*53^−/−^ lung organoids and 2D culture. Lung of C57BL/6 J mouse was isolated and processed as described in the Methods. **B** Representative bright field, H&E and IHC staining for EpCAM, SFTPC of the normal organoids. Scale bars: 50 μm. **C** Representative merged, EGFP and bright field channel for control organoid and EGFP + organoid. Scale bars: 50 μm. **D** Schematic of the strategy for early LUAD cells underwent an “in vivo Cycle” and in vitro cultured cells as control. **E** Schematic for the timeline of total three rounds of “in vivo Cycle” and relative in vitro control. **F** Orthotopic pulmonary injection tumor formation assays result of AP and AHS group cells. The red arrows indicate the tumor region. **G** Representative H&E and IHC staining for SFTPC and p63 of AHS-T4 in situ lung tumor. The orange dot line indicates the boundary of tumor and normal lung tissues. T: Tumor; L: Lung. Scale bars: 100 μm

To investigate the initiation and evolution of early lung adenocarcinoma while preserving native cell–cell interactions, we established murine lung organoids from *Kras*^*LSL−G12D/*+^*/Trp53*^*fl/fl*^ C57BL/6 J mice (Fig. 1A). Immunohistochemical staining confirmed the expression of EpCAM and SFTPC (a marker for alveolar type II cells), indicating that the organoids recapitulated the cellular origins of LUAD (Fig. 1B). We then introduced Kras G12D activation and Trp53 deletion via lentiviral delivery of Cre-EGFP (Fig. 1A, C). Genotyping of EGFP^+^ murine lung organoids confirmed successful mutation introduction (Figure S1A). For subsequent functional assays, EGFP^+^ organoids were adapted to 2D culture to facilitate rapid construction of corresponding murine models for downstream experiments (Figure S1B).

To determine whether the in vivo immune microenvironment drives immune evasion in early tumor cells following oncogenic transformation, we designed the following experiment using 2D-cultured tumor cells derived from EGFP⁺ organoids (Fig. 1D, E): We defined the baseline time point “T1” as representing naïve early LUAD cells before any in vivo exposure; these are referred to as “AP-T1” cells. AP-T1 cells were orthotopically injected into the left lung lobe to expose them to pulmonary immune selective pressure. To avoid rapid clearance of highly immunogenic naive tumor cells, we optimized a protocol wherein injected cells were recovered 48 h post-injection. EGFP⁺ cells were then enriched, expanded, and analyzed. This process-from injection to recovery and expansion-was designated one “in vivo Cycle” (Figure S1C). Cells recovered after one cycle (AHS-T2) were re-injected for subsequent cycles, yielding AHS-T3 and AHS-T4 samples (in vivo group, AHS, Figure S1D, E). In parallel, AP-T1 cells were passaged in vitro for equivalent durations, generating control samples AP-T2, AP-T3, and AP-T4 (in vitro group, AP).

Following the harvest of the seven cell lines, we characterized their proliferative capacity and malignant potential. CCK-8 assays revealed that both AP and AHS groups exhibited increased proliferation rates over successive time points (Figure S2A). Notably, at any given time point (e.g., AP-T2 vs. AHS-T2), cells from both groups displayed comparable growth behaviors and consistent proliferative potentials (Figure S2B). These findings were further corroborated by plate colony formation assays (Figure S2C-D). To further evaluate malignant transformation of cells, we performed the anchorage independent growth assay in soft agar. While AP-T1, AP-T2, and AHS-T2 cells failed to form detectable colonies, both AP and AHS cells at the T3 and T4 stages generated significant colony formation. At these later timepoints, the AHS group exhibited a markedly higher clonogenic capacity than the corresponding AP group at the same timepoint (i.e., AHS-T4 > AP-T4). Additionally, within each group, T4 cells showed greater colony-forming efficiency than T3 cells (Figure S2E, F). In summary, these results demonstrate that while both groups gained proliferative and malignant potential over time, the AHS group acquired a significantly enhanced malignant phenotype following iterative in vivo immune selection.

To evaluate differences in tumorigenic potential and immune evasion between in vivo-selected and in vitro-cultured cells, we performed lung orthotopic implantation of AP group and AHS group cells into mice (Fig. 1F). Results demonstrated that only AHS-T4 cells successfully formed detectable orthotopic pulmonary tumors in all five injected mice. AHS-T3 cells produced one single detectable nodule in one mouse, while all other cell lines (AP-T1–T4 and AHS-T2) failed to establish visible tumors (Fig. 1F). Histopathological and immunohistochemical analyses confirmed tumor formation and expression of LUAD markers SFTPC and P63 (Fig. 1G). These results indicate that AHS-T4 cells, after undergoing selection in the native immune microenvironment, acquired enhanced tumorigenic and immune-evasion capabilities compared to their in vitro-cultured counterparts.

### HOXA10 is induced in early-stage LUAD

To investigate the progressive genomics alterations that facilitate LUAD progression in vivo, we performed whole-exome sequencing on cells from all seven time points of our mouse tumor model (Fig. 1E, Figure S3A, B). Analysis of copy number variation (CNV) showed a time-dependent increase in the number of genes affected by CNVs in AHS samples (Figure S3C). In contrast, AP samples displayed minimal change in CNV burden over time, with no evidence of progressive accumulation (Figure S3D). Notably, the tumor suppressor genes, *Arid1a* and *Rb1,* which showed frequently loss in human LUAD, was detected in our model, underscoring its clinical relevance (Figure S3E) [30–32]. Mutational analysis showed a gradual increase in the number of genetic variants over time, with AHS samples consistently harboring more mutations than their AP counterparts. The top 10 most frequently mutated genes included known human LUAD-associated genes such as *Ttn* and *Tmprss2* (Figure S3F) [33–35].

**Fig. 2:**
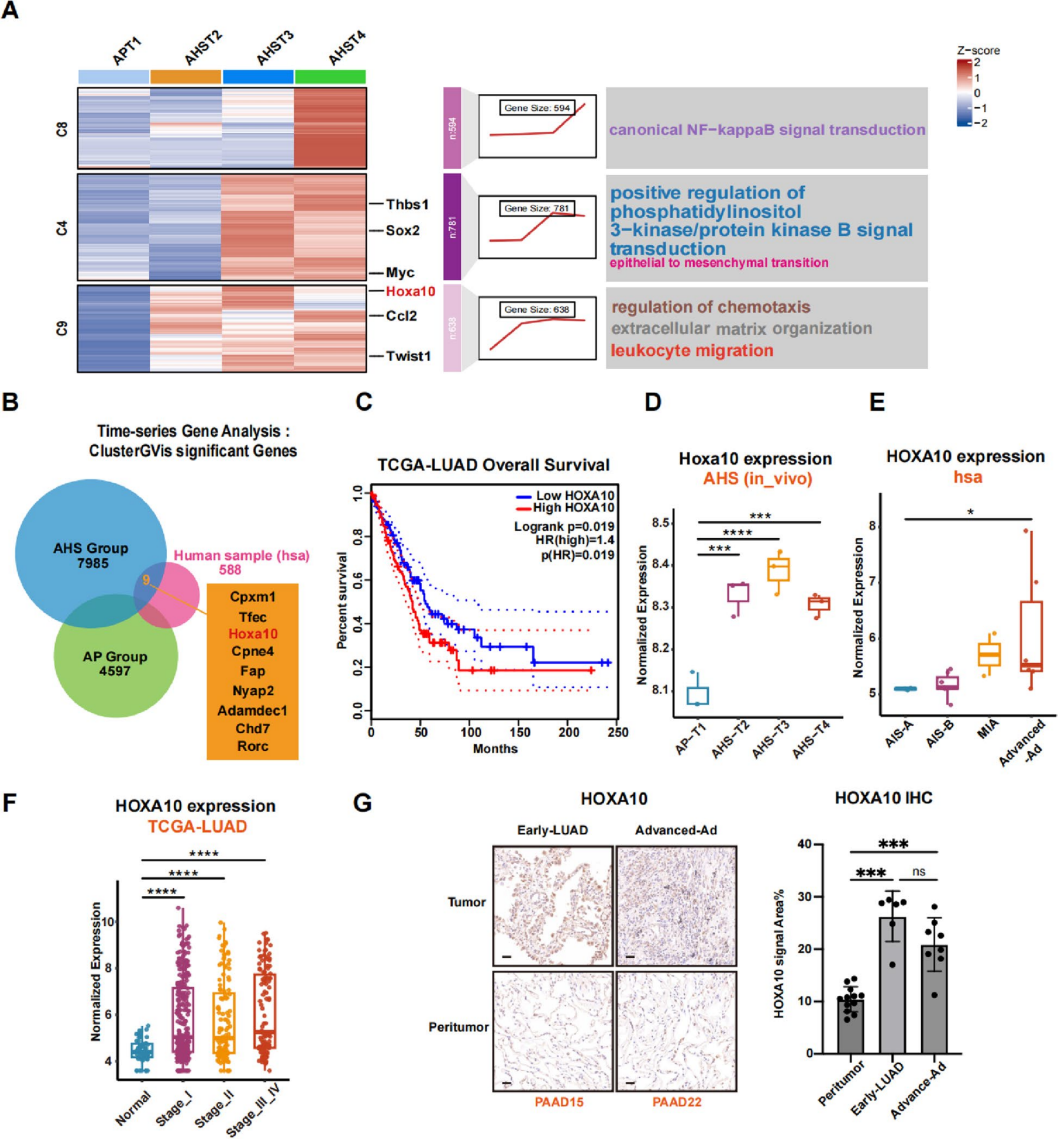
HOXA10 is upregulated in early-stage LUAD. **A** Three major clusters of Time-series Gene Expression Analysis (TGEA) for AHS groups. **B** Venn diagram for TGEA significant genes of AHS, AP and hsa group. **C** Overall survival of TCGA LUAD samples. The samples were divided into two groups by HOXA10 expression level: High HOXA10: *n* = 239, Low HOXA10: *n* = 237. **D** Normalized Hoxa10 expression level of AHS group RNAseq data. Data were presented as mean ± SD. Each group *n* = 3. **E** Normalized HOXA10 expression level of Human sample (hsa) RNAseq data. AIS-A:*n* = 3; AIS-B:*n* = 7; MIA:*n* = 2; Advanced-Ad:*n* = 6. Data were presented as mean ± SD. **F** Normalized HOXA10 expression level of TCGA-LUAD RNAseq data. Data were presented as mean ± SD. Normal (*n* = 59), Stage I (*n* = 288), Stage II (*n* = 124), Stage III (*n* = 84), Stage IV (*n* = 26). **G** Representative IHC staining for HOXA10 of early stage and advanced LUAD tumor samples with paired peritumor tissues. HOXA10 expression level are quantified for three groups. Data were presented as mean ± SD. Peritumor *n* = 12, Early-LUAD *n* = 6, Advanced-Ad *n* = 8. *p < 0.05, **p < 0.01, ***p < 0.001, ****p < 0.0001, ns: not significant

Next, we performed RNA sequencing on cells from all seven time points to compare temporal gene expression differences between the AHS and AP groups. Principal component analysis (PCA) showed clear separation between AHS and AP groups, indicating pronounced transcriptional differences between these two conditions (Figure S4A). We then conducted Time-series Gene Expression Analysis (TGEA) to identify genes whose expression patterns significantly correlated with temporal progression (Figure S4B, C, Table S1, S2). Functional enrichment analysis based on GO showed that genes involved in NF-κb signal transduction, regulation of chemotaxis, and extracellular matrix organization pathways were gradually upregulated in AHS samples (Fig. 2A). To align our findings with clinical relevance, we performed parallel analyses using RNA-seq data from a cohort of early-stage LUAD patients (designated as the “hsa group”), which were categorized into four subgroups based on disease stage: Adenocarcinoma in situ types A and B (AIS-A, AIS-B), Minimally Invasive Adenocarcinoma (MIA), and Advanced Adenocarcinoma (Advanced-Ad) (Figure S4D, E) [36]. Notably, AIS-A/B and MIA represent very early lesions that are typically associated with favorable prognosis. By integrating TGEA results from AHS, AP, and hsa groups, we identified genes showing consistent expression patterns in both the AHS and clinical cohorts, but not in the AP group. This filtering strategy yielded nine candidate genes potentially associated with the acquisition of immune evasion in early LUAD (Fig. 2B). Survival analyses of the nine candidate genes using TCGA-LUAD patient data revealed that HOXA10, a homeobox transcription factor which has been reported to promote malignant progression in multiple cancers [8, 9], was the only gene with significant prognostic value (Fig. 2C, Figure S4F). Therefore, we chose HOXA10 for further investigation.

Importantly, we observed progressive upregulation of Hoxa10 expression over time in both murine AHS group (Fig. 2D) and the early-stage clinical LUAD cohort (Fig. 2E) while expression remained unchanged in AP samples (Figure S4G). Consistent with these findings, Analysis of The Cancer Genome Atlas (TCGA) LUAD RNA-seq data showed that HOXA10 was significantly upregulated as early as stage I compared to normal samples, with sustained high expression maintained throughout stages II-IV (Fig. 2F).

To further validate HOXA10 upregulation in LUAD, we collected pathological specimens of clinical LUAD patients (Table S3), including early-stage (Early-LUAD), advanced-stage (Advanced-Ad), and adjacent normal control samples (Peritumor), and performed immunohistochemical staining to quantify HOXA10 expression (Fig. 2G). Results demonstrated that, consistent with our previous findings, HOXA10 exhibited elevated expression in early-stage LUAD patient tissues and maintained high expression levels in advanced-stage samples. Collectively, these findings suggest that HOXA10 upregulation is a critical event during the early development of lung adenocarcinoma and is associated with the acquisition of immune evasion potential by tumor cells.

Given HOXA10’s established role in epithelial-mesenchymal transition (EMT) [17], we evaluated EMT scores across our cell lines using the transcriptomic data. Notably, the AP group exhibited a progressive decline in EMT scores over time, whereas the AHS group showed a rapid and steady increase (Figure S5A). RNA-seq analysis of core markers corroborated this divergence: the mesenchymal markers *Cdh2* (N-cadherin) and *Vim* (Vimentin) were significantly upregulated in AHS cells but declined or remained low in the AP group (Figure S5B, D). Conversely, the epithelial marker *Cdh1* (E-cadherin) remained high in AP cells but markedly decreased in the AHS group (Figure S5C).

We further validated these transcriptomic shifts at the protein level via Western blotting. As shown in Figure S5E, AHS cells exhibited significantly higher N-cadherin and lower E-cadherin levels than time-matched AP cells. Notably, Vimentin expression was detected exclusively in the AHS group. Collectively, these findings demonstrate that iterative in vivo selection significantly enhances the EMT potential of AHS cells.

### HOXA10 depletion impairs tumorigenesis and enhances anti-tumor immunity

**Fig. 3:**
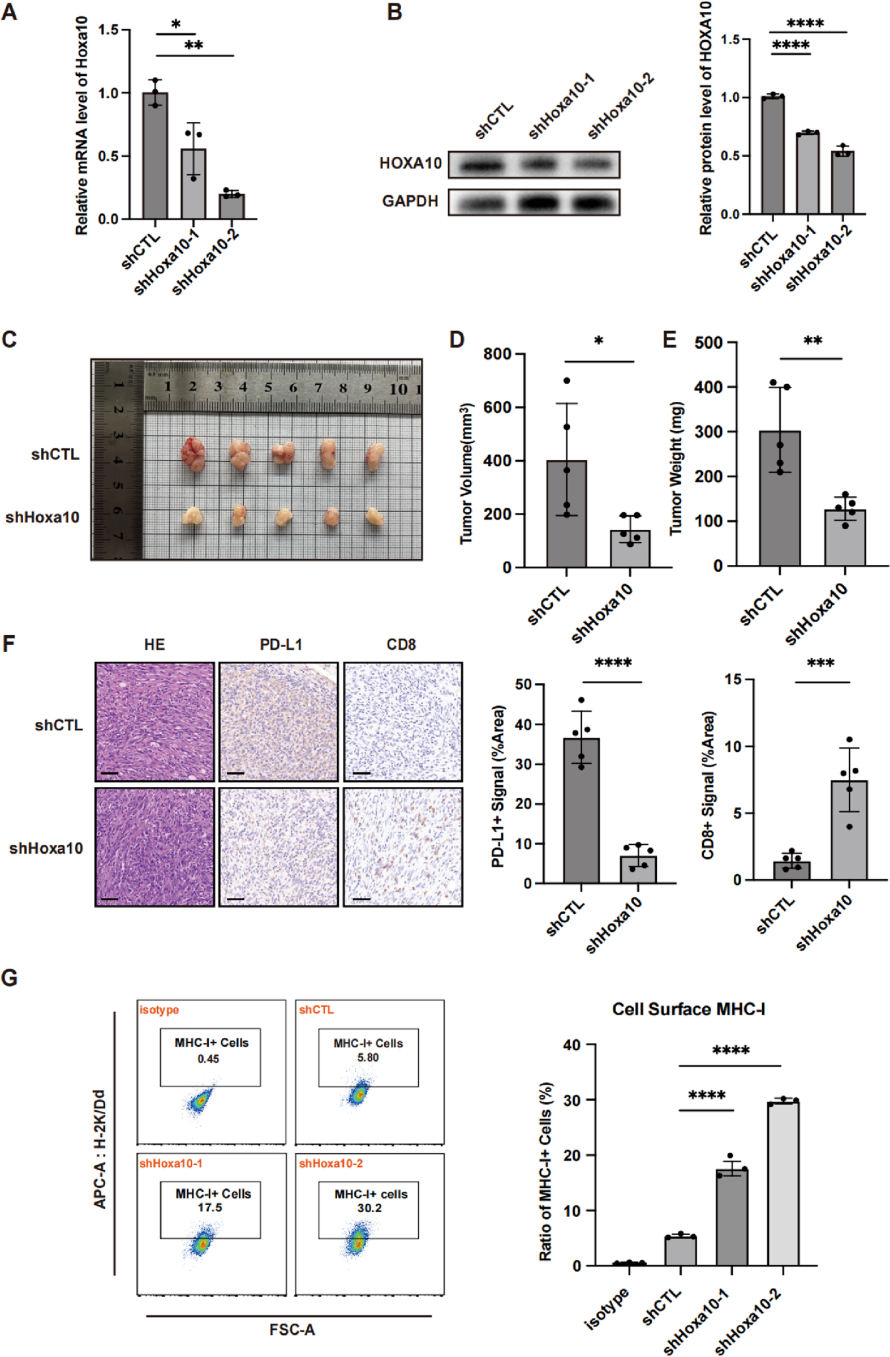
HOXA10 depletion impairs tumorigenesis and enhances anti-tumor immunity. **A**-**B** Knockdown of Hoxa10 in AHS-T4 Cells were verified by RT-qPCR (**A**) and Western Blot (**B**). *n* = 3. Data were presented as mean ± SD. **C**-**E** The subcutaneous tumor of shCTL and shHoxa10 group (**C**). Tumor volume (**D**) and tumor weight (**E**) were measured. *n* = 5 each. Data were presented as mean ± SD. **F** Representative H&E and IHC staining for PD-L1 and CD8 of subcutaneous tumors. *n* = 5 for each group. Data were presented as mean ± SD. Scale bars: 50 μm. **G** Flow Cytometry analysis for H-2 K/Dd (MHC-I) of shCTL and shHoxa10-1, shHoxa10-2 Cells. *n* = 3 each. Data were presented as mean ± SD. *p < 0.05, **p < 0.01, ***p < 0.001, ****p < 0.0001, ns: not significant

To investigate the potential oncogenic mechanisms of HOXA10, we generated Hoxa10 knockdown cell lines (shHoxa10-1, shHoxa10-2) from AHS-T4 cells and confirmed the reduction of HOXA10 at both the mRNA (Fig. 3A) and protein levels (Fig. 3B). We then assessed the tumorigenic capacity of these cells using both subcutaneous and lung orthotopic tumor model. Compared with control shCTL cells, Hoxa10 knockdown significantly suppressed tumor growth in both models (Fig. 3C-E, Figure S6A, B). To determine whether Hoxa10 depletion alters the tumor immune microenvironment, we performed immunohistochemical staining on the harvested subcutaneous tumors. Assessment of overall CD8 and PD-L1 expression within tumors revealed significantly stronger CD8 signals in the shHoxa10 group, accompanied by relatively weaker PD-L1 signals (Fig. 3F). These findings suggest that Hoxa10 knockdown may enhance CD8^+^ T cell infiltration and establish a more immunologically active tumor microenvironment. We further examined tumor-intrinsic changes using flow cytometry to quantify surface MHC class I (H-2 K/Dd) expression. This analysis revealed that both shHoxa10-1 and shHoxa10-2 cells exhibited significantly elevated surface MHC class I levels compared to control cells (Fig. 3G). These findings collectively indicated that HOXA10 upregulation in early lung adenocarcinoma may facilitate immune evasion and fostering an immunosuppressive microenvironment, thereby promoting tumor survival and growth.

### Hoxa10 knockdown remodels the tumor immune microenvironment

**Fig. 4:**
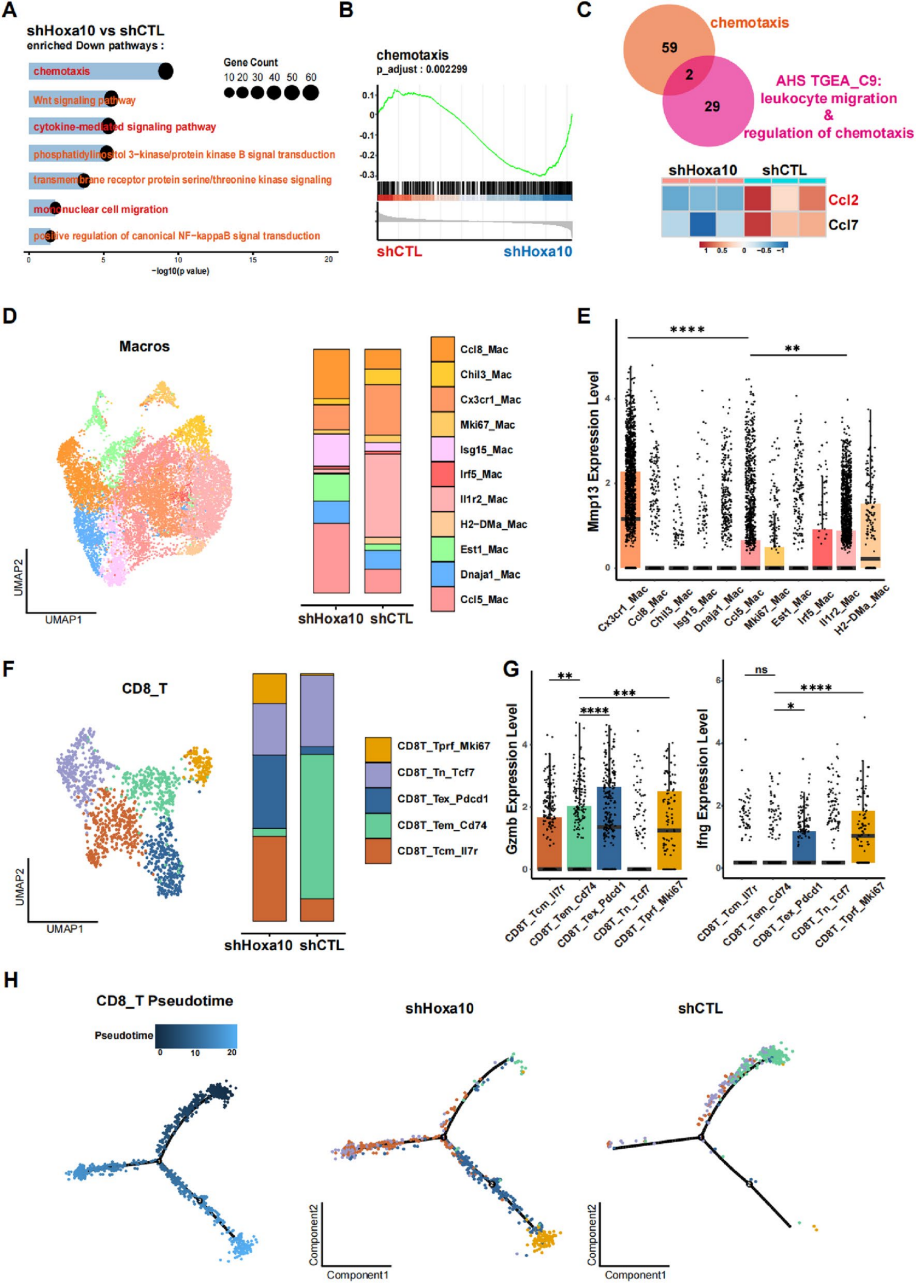
Hoxa10 depletion reshapes the tumor immune microenvironment. **A** RNAseq in shCTL and shHoxa10. Significant down-regulated pathways in GO analysis. **B** GSEA for chemotaxis gene sets. **C** Venn diagram and heatmap of intersected genes of “chemotaxis”, “regulation of chemotaxis” and “leukocyte migration” pathways. **D** UMAP (Uniform manifold approximation and projection) plot for subtypes of macrophages. Macros: macrophages. Frequency of each cell subtypes are presented (right). **E** Mmp13 expression level of each macrophages cell subtypes. Data were presented as mean ± SD. **F** UMAP plot for subtypes of CD8 + T cells. CD8_T: CD8^+^ T Cell. Frequency of each cell subtypes are presented (right). **G** Gzmb and Ifng expression level of each CD8 + T cell subtypes. Data were presented as mean ± SD. **H** Pseudotime analysis of CD8 + T cells of each Group. *p < 0.05, **p < 0.01, ***p < 0.001, ****p < 0.0001, ns: not significant

Given the critical role of HOXA10 in tumor immune evasion, we subjected shCTL and Hoxa10-deficient AHS-T4 tumors for RNA sequencing to elucidate the mechanisms underlying Hoxa10-mediated pro-tumoral immunity. A total of 3,205 differentially expressed genes were identified, including 1,788 downregulated and 1,417 upregulated genes in shHoxa10 tumors compared with shCTL tumors (Figure S7A, Table S4). GO analysis revealed significant downregulation of pathways such as chemotaxis, cytokine-mediated signaling pathway, and mononuclear cell migration in the knockdown group (Fig. 4A). Gene Set Enrichment Analysis (GSEA) further confirmed a strong enrichment of chemotaxis in shCTL tumors (Fig. 4B), suggesting that HOXA10 may modulates the immune microenvironment through regulation of monocyte-associated chemokine signaling. Notably, in our previous TGEA, GO terms for cluster_9 (which includes Hoxa10)-such as “regulation of chemotaxis” and “leukocyte migration”-were also significantly enriched (Fig. 2A), reinforcing the link between HOXA10 and immune cell recruitment. To pinpoint key chemokines involved, we intersected genes from the “chemotaxis”, “regulation of chemotaxis”, and “leukocyte migration” pathways and identified Ccl2 and Ccl7 as candidates showing progressive upregulation during cancer progression. Both genes were expressed at significantly higher levels in shCTL cells, with Ccl2 exhibiting the most marked increase (Fig. 4C). Given that CCL2 is known to recruit immunosuppressive macrophages in cancer contexts [37, 38], we hypothesized that HOXA10 may facilitate the chemotactic recruitment of immunosuppressive macrophages through upregulation of CCL2 expression.

To comprehensively examine the effect of HOXA10 on tumor immune microenvironment, we performed single-cell RNA sequencing on immune cells isolated from subcutaneous tumors of shCTL and shHoxa10 groups. We annotated major immune cell populations based on classical markers for key immune cell types (Figure S7B). Comparative analysis revealed that macrophages (Macros) and T cells (CD8_T, CD4_T) constituted the predominant immune subsets. Notably, the shHoxa10 group exhibited a decreased proportion of macrophages and a concomitant increase in CD8⁺ and CD4⁺ T cells compared to the shCTL group (Figure S7C). We next conducted subset-specific analyses of macrophages, CD8⁺ T cells, and CD4⁺ T cells. Macrophages were independently reclustered and subclassified into distinct transcriptional subsets (Figure S7D), and the proportional abundance of each subtype was compared between conditions (Fig. 4D). We observed an increased proportion of M1-like macrophages, including Ccl8_Mac, Isg15_Mac, and Ccl5_Mac, which highly express immune activating genes such as Cd80, Cd86 and Tnf in the shHoxa10 group. In contrast, shCTL tumors were enriched in M2-like macrophages, including Chil3_Mac, Cx3cr1_Mac, and Il1r2_Mac cells, which exhibit elevated expression of immunosuppressive markers including Cx3cr1, Spp1, Chil3 and Il1r2. Additionally, Cx3cr1_Mac and Il1r2_Mac exhibited significantly elevated expression of Mmp13, Mmp19 and Fn1 compared to Ccl5_Mac (Fig. 4E, Figure S7E). Elevated expression of these genes is typically associated with tumor-associated extracellular matrix remodeling and impedance of immune cell infiltration [39–41].

We conducted similar analyses to CD8⁺ and CD4⁺ T cell subsets (Fig. 4F, Figure S7F, H). In the shHoxa10 group, we observed a marked increase in proliferating (CD8T_Tprf_Mki67), activated exhausted (CD8T_Tex_Pdcd1), and central memory (CD8T_Tcm_Il7r) CD8⁺ T cell populations. In contrast, CD8^+^ T cells in the shCTL group were primarily composed of naïve (CD8T_Tn_Tcf7) and effector memory (CD8T_Tem_Cd74) subsets (Fig. 4F). Notably, CD8T_Tprf_Mki67 and CD8T_Tex_Pdcd1 cells exhibited significantly elevated expression of cytotoxic mediators including Gzmb, Ifng, and the perforin gene Prf1 compared to CD8T_Tem_Cd74 cells (Fig. 4G, Figure S7G), suggesting enhanced tumor-killing and immune activation capabilities within the shHoxa10 tumors. Pseudotime trajectory analysis of CD8⁺ T cell subtypes further revealed distinct differentiation patterns between the groups: CD8⁺ T cells from shCTL tumors clustered near the origin of the trajectory, whereas those from shHoxa10 tumors were enriched along two later branches, indicating more advanced priming and differentiation (Fig. 4H). Similarly, among CD4⁺ T cell subsets, we detected significant enrichment of CD4T_Th1-like cells in shHoxa10 tumors (Figure S7H, I), consistent with a broad shift toward immune activation in the absence of HOXA10.

Since macrophages and CD8⁺ T cells represent major immune constituents in both tumor groups, and considering prior evidence that M1-type macrophages can enhance CD8^+^ T cell cytotoxicity through direct and indirect interactions [41–43], we hypothesized that macrophage-CD8^+^ T cell crosstalk may play a pivotal role in reshaping the immune microenvironment. To explore this, we analyzed cell–cell interaction networks and identified strong potential interactions between Isg15_Mac/Ccl5_Mac populations and CD8T_Tprf_Mki67/CD8T_Tex_Pdcd1 subsets, along with CD80- and CD86-mediated T cell co-stimulatory signals (Figure S7J), suggesting a likely mechanism for enhanced T cell activation in shHoxa10 tumors.

### Hoxa10 deficiency reprograms the TME toward an immunoactivating state

To evaluate the comprehensive impact of HOXA10 on the tumor immune microenvironment, we performed multiplex Immunohistochemical (mIHC) staining on both subcutaneous and orthotopic lung tumor sections derived from shCTL and shHoxa10 cell lines. Our panel included CD4, CD8, FOXP3, GZMB, F4/80, and PD-L1, allowing for the simultaneous identification of major immune subsets and the assessment of overall immune activation status [44, 45].

In the subcutaneous model (Figure S8A), we observed substantial infiltration of CD8 + T cells and F4/80 + macrophages. Consistent with our scRNA-seq findings (Figure S7C), shHoxa10 tumors exhibited significantly stronger CD8 signals and attenuated F4/80 signals compared to the shCTL group. While CD4 + T cell infiltration remained low in both groups, the immunosuppressive FOXP3 + Treg signal was markedly more prominent in shCTL tumors. Furthermore, the elevated GZMB expression and reduced PD-L1 levels in shHoxa10 sections indicate a more robust anti-tumor immune response and a shift toward an immunostimulatory TME. These findings were further validated in the orthotopic lung tumor model, which displayed a strikingly similar immune landscape (Figure S8C).

By analyzing the co-localization of CD8 and GZMB, we evaluated the infiltration of CD8 + GZMB + cytotoxic T cells. As illustrated in Figure S8B and D (orange circles indicating co-localization), shHoxa10 tumors showed a dramatic increase in cytotoxic T cell recruitment in both subcutaneous and orthotopic settings. Collectively, these results demonstrate that inhibiting tumor-intrinsic HOXA10 expression reprograms the TME toward an immunoactivating state.

### Hoxa10 promotes an immunosuppressive tumor microenvironment via CCL2

**Fig. 5:**
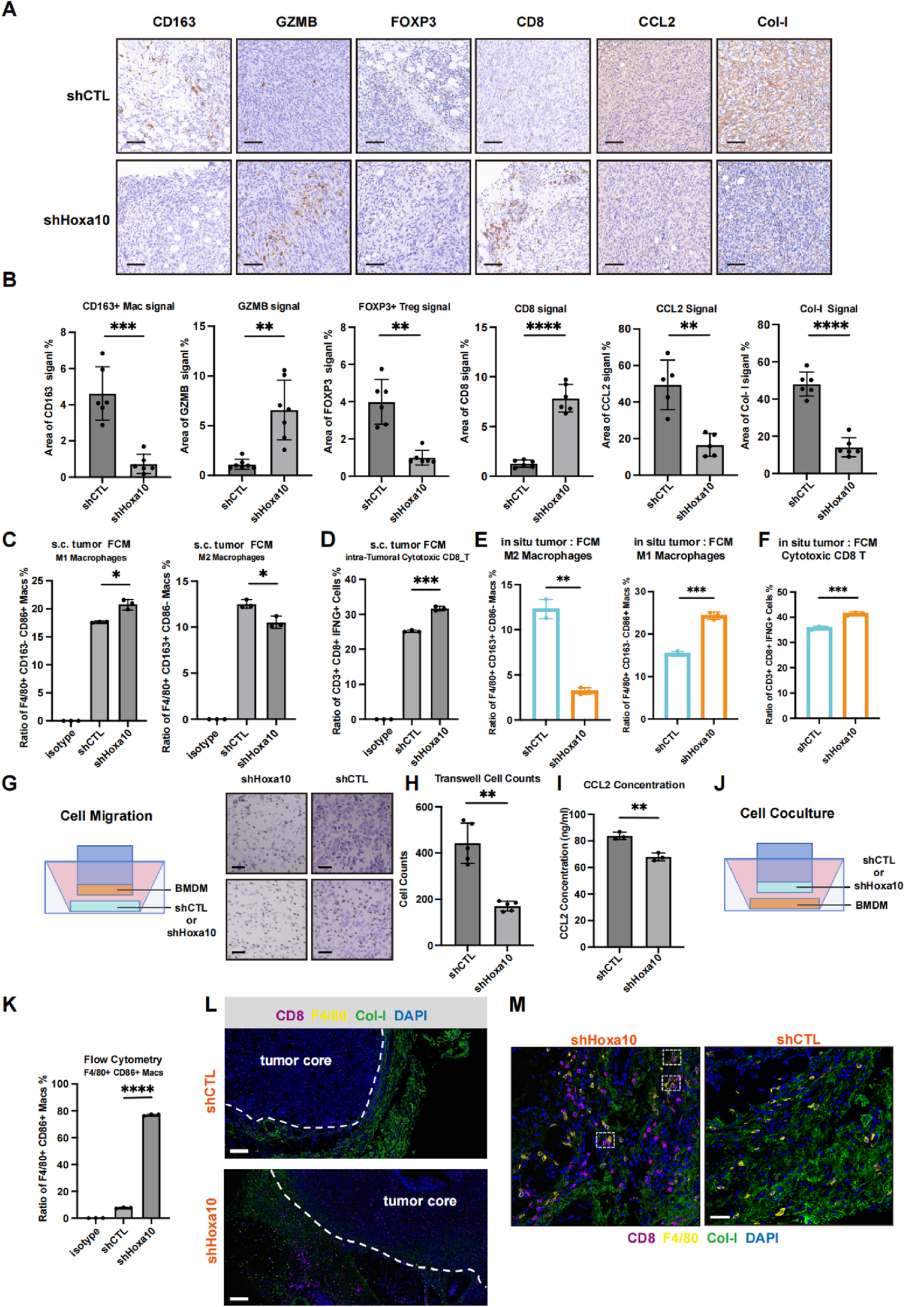
Hoxa10 sustains an immunosuppressive microenvironment via CCL2. **A**-**B** Representative IHC staining and quantification for CD163 (*n* = 6), GZMB (*n* = 7), FOXP3 (*n* = 6), CD8 (*n* = 6), CCL2 (*n* = 5), Col-I (*n* = 6) of shCTL and shHoxa10 group subcutaneous tumors. Data were presented as mean ± SD. Scale bars: 50 μm. **C** Flow Cytometry analysis for F4/80, CD86, CD163 of macrophages in subcutaneous tumors. Data were presented as mean ± SD. *n* = 3. **D** Flow Cytometry analysis for CD3, CD8, IFNG of T cells in subcutaneous tumors. *n* = 3. Data were presented as mean ± SD. **E** Flow Cytometry analysis for F4/80, CD86, CD163 of macrophages in orthotopic pulmonary tumors. Data were presented as mean ± SD. *n* = 3. **F** Flow Cytometry analysis for CD3, CD8, IFNG of T cells in orthotopic pulmonary tumors. *n* = 3. Data were presented as mean ± SD. **G**-**H** Schematic and representative images of migration assays of BMDM (**G**). Cell counts are quantified (**H**). *n* = 5. BMDM: Bone Marrow derived Macrophages. Scale bars: 30 μm. Data were presented as mean ± SD. **I** ELISA assays of CCL2 secretion of shCTL and shHoxa10 Cell in migration assays. *n* = 3. Data were presented as mean ± SD. **J** Schematic of indirect cell co-culture. **K** Cytometry analysis for F4/80, CD86 of co-cultured BMDMs. *n* = 3. Data were presented as mean ± SD. **L** Representative multiplex Immunohistochemical staining for CD8, F4/80, Col-I, DAPI of subcutaneous tumor sections. The white dot lines indicate the boundary of tumor. Scale bars: 200 μm. **M** Representative view of “Macrophages-CD8^+^ T Cell” niches indicated by white dashed boxes in shHoxa10 group subcutaneous tumors. Scale bars: 100 μm. *p < 0.05, **p < 0.01, ***p < 0.001, ****p < 0.0001, ns: not significant

To further validate the above findings, we conducted the following experiments. Immunohistochemical staining and quantitative analysis of subcutaneous tumor sections from shCTL and shHoxa10 mice revealed that the shHoxa10 group exhibited significantly elevated cytotoxic-related signals marked by GZMB and CD8, along with markedly reduced signals for CD163^+^ M2 macrophages, FOXP3^+^ regulatory T cells (Tregs), Col-I-marked TIB remodeling and CCL2, consistent with the single-cell sequencing results (Fig. 5A, B). Subsequent flow cytometry analysis showed a significant increase in M1 macrophages (F4/80⁺ CD163⁻ CD86⁺ cells) and a decrease in M2 macrophages (F4/80⁺ CD163⁺ CD86⁻ cells) within shHoxa10 tumors (Fig. 5C, Figure S9A, B). Similarly, flow cytometric profiling of tumor-infiltrating T cells showed a pronounced increase in the proportion of CD3⁺ T cells (Figure S9A, C) and, notably, a higher percentage of CD3⁺ CD8⁺ IFN-γ⁺ cytotoxic T cells in the shHoxa10 group (Fig. 5D, Figure S9A, D). We further conducted immunohistochemical staining (Figure S9E-F) and flow cytometry analysis (Fig. 5E-F, Figure S9G) using the orthotopic tumor model, and the results were highly consistent with those of the subcutaneous tumor models.

To further investigate the effects of tumor cell Hoxa10 expression on macrophages behavior, we employed in vitro co-culture systems. Migration assays using bone marrow-derived macrophages (BMDMs) co-cultured with shCTL or shHoxa10 cells demonstrated significantly impaired macrophage migration capacity in the shHoxa10 group (Fig. 5G, H). ELISA analysis of cell culture supernatants showed markedly reduced CCL2 secretion from shHoxa10 cells (Fig. 5I), whereas Hoxa10-overexpressing cells (Hoxa10-OE) showed elevated CCL2 levels (Figure S9H). Furthermore, flow cytometry analysis showed a significant increase in the proportion of F4/80⁺ CD86⁺ macrophages following co-culture with shHoxa10 cells (Fig. 5J, K, Figure S9I-J), suggesting that HOXA10 promotes the recruitment of immunosuppressive macrophages.

To validate whether Hoxa10 knockdown enhances interactions within macrophage-CD8⁺ T cell niches, we performed multiplex Immunohistochemical staining on subcutaneous tumor sections from both mouse groups. The results demonstrated a marked increase in overall CD8 signal intensity in shHoxa10 tumors, while Col-I-marked TIB structures were more prominent and displayed a dense reticular network pattern in the shCTL group (Fig. 5L), consistent with previous studies that such physical barriers hinder immune cell infiltration [40]. Although F4/80⁺ macrophages in both groups localized predominantly along Col-I-rich tumor margins, frequent co-localization of F4/80 and CD8 signals was observed specifically in shHoxa10 tumor (Fig. 5M, dashed boxes), a pattern absent in the shCTL group. These findings indicate that Hoxa10 knockdown promotes the formation of macrophage-CD8⁺ T cell niches within the TME, which may facilitate enhanced anti-tumor efficacy of CD8⁺ T cells and other immune effector cells.

### HOXA10 promoted CCL2 secretion by upregulating canonical NF-κB signaling

**Fig. 6:**
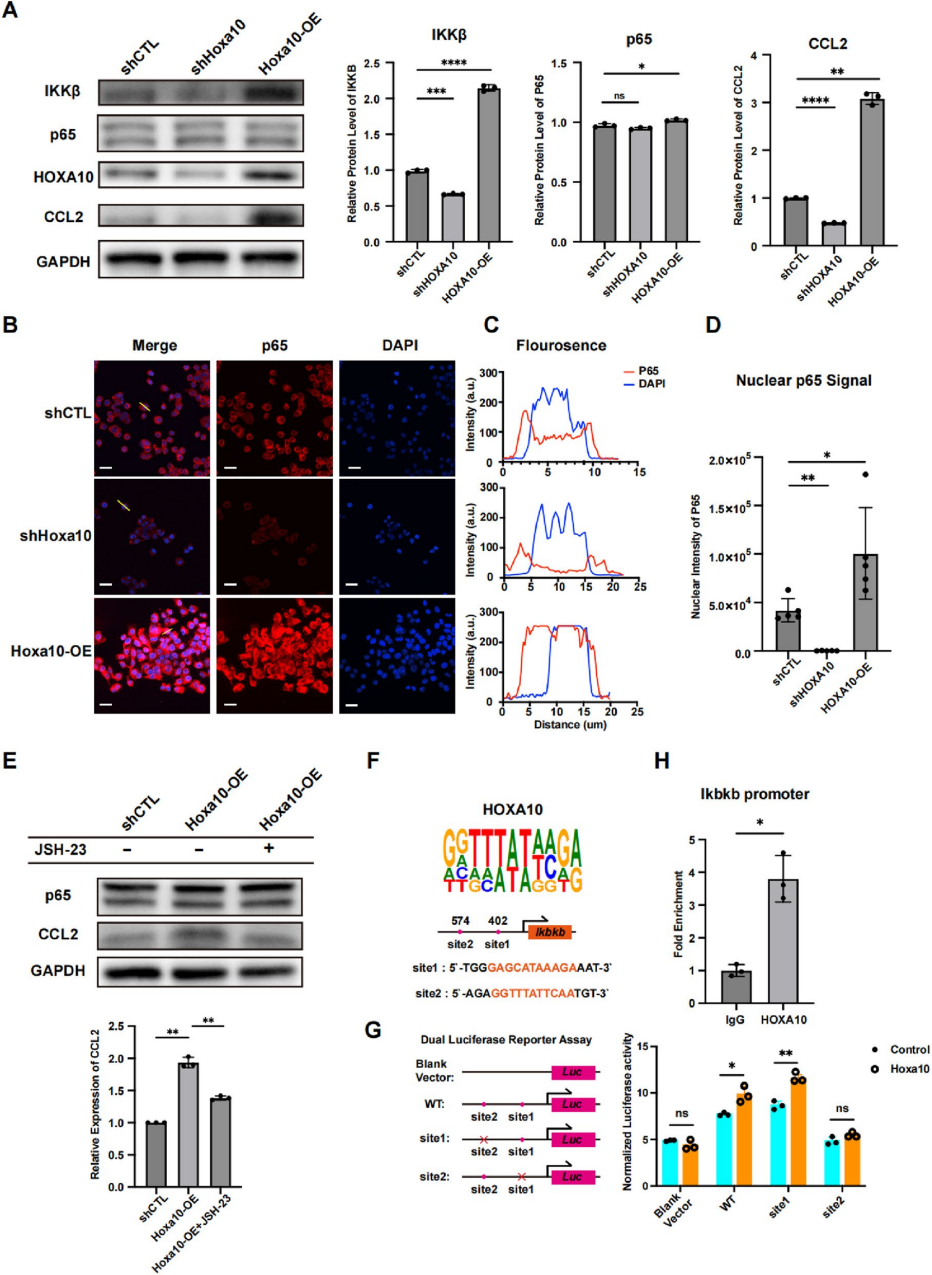
HOXA10 promoted CCL2 secretion by upregulating canonical NF-κB signaling. **A** Western Blot analysis of IKKB, p65, HOXA10, CCL2 expression level in shCTL, shHoxa10 and Hoxa10-OE Cells. Hoxa10-OE: Hoxa10 overexpression cell. *n* = 3. Data were presented as mean ± SD. **B** Representative immunofluorescence staining for p65 and DAPI. Scale bars: 20 μm. **C** Representative fluorescence intensity in cell which is indicated by yellow lines in (**B**). **D** Quantification of nuclear p65 intensity in cells. *n* = 5. Data were presented as mean ± SD. **E** Western Blot analysis of p65 and CCL2 expression level of Hoxa10-OE cells treated with JSH-23. CCL2 protein level are quantified. *n* = 3. Data were presented as mean ± SD. **F** Schematic of predicted HOXA10 binding motifs to Ikbkb promoter region. **H** RT-qPCR analysis for Ikbkb promoter region enrichment of ChIP assay. **G** Dual luciferase reporter assay for binding site verification. The red cross means mutant of that binding site. “Hoxa10” means Hoxa10 overexpression. WT: wild type. *n* = 3. Data were presented as mean ± SD. *p < 0.05, **p < 0.01, ***p < 0.001, ****p < 0.0001, ns: not significant

CCL2, a key chemokine, is primarily regulated through the canonical NF-κB signaling pathway [46, 47]. In conjunction with the Time-series Gene Expression Analysis of AHS group cells, we observed concurrent upregulation of both Hoxa10 expression and canonical NF-κB signaling (Fig. 2A). We therefore investigated whether Hoxa10 influences the expression of core components of this pathway, namely IKKβ and p65. Hoxa10 knockdown significantly reduced the expression of IKKβ and CCL2, whereas Hoxa10 overexpression substantially upregulated both, although p65 expression remained largely unchanged (Fig. 6A, Figure S10A, B). Since nuclear translocation of the p65 protein is a hallmark of canonical NF-κB activation [48], we evaluated p65 localization via immunofluorescence. We observed a pronounced decrease in nuclear p65 intensity in shHoxa10 cells, and a marked increase in Hoxa10-OE cells, within DAPI-defined nuclear regions (Fig. 6B-D). To further assess the impact of p65 nuclear translocation on CCL2 expression, we treated Hoxa10-OE cells with JSH-23, an inhibitor of p65 nuclear import. This treatment significantly attenuated the elevated CCL2 levels observed in Hoxa10-OE cells (Fig. 6E). These results suggest that HOXA10 may modulates CCL2 expression by regulating IKKβ protein levels, thereby affecting P65 activation and its nuclear translocation.

We further investigated how IKKβ expression was regulated by HOXA10. Through bioinformatics prediction, we identified two potential Hoxa10 binding sites within the promoter region of the Ikbkb gene (encoding IKKβ) (Fig. 6F). A dual-luciferase reporter system demonstrated that deletion of site1 abolished the transcriptional activation of the Ikbkb promoter by HOXA10. This result was further confirmed by ChIP-qPCR analysis, which showed specific enrichment of HOXA10 at this promoter region (Fig. 6G-H). Collectively, these findings indicated that HOXA10 transcriptionally activates Ikbkb expression, thereby modulating CCL2 production through the canonical NF-κB signaling pathway.

### Inhibiting HOXA10 boosts anti-PD-L1 therapy effects

**Fig. 7:**
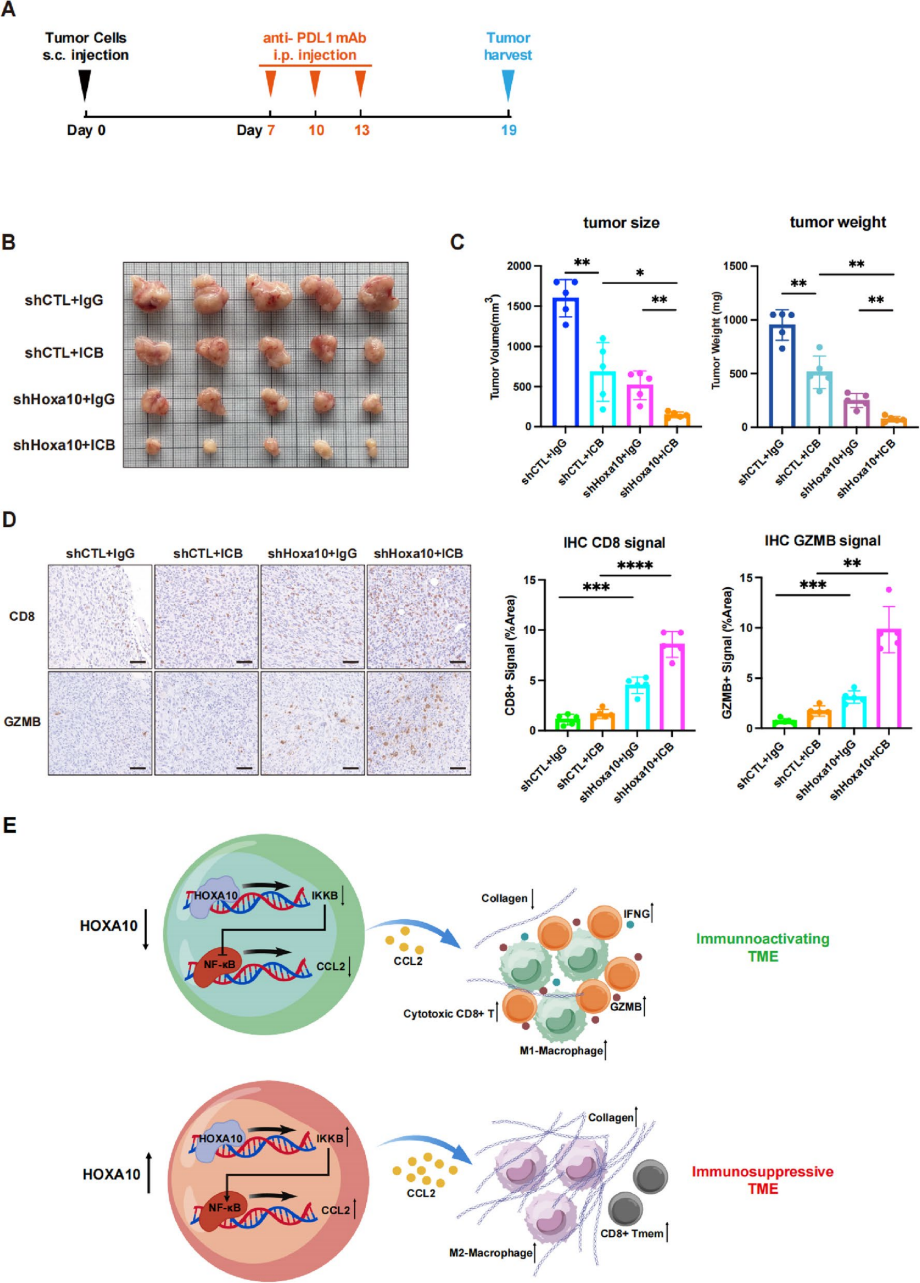
Inhibiting HOXA10 boosts anti-PD-L1 therapy effects. **A** Schematic representation of the experimental procedure. **B**-**C** The subcutaneous tumor of each group. Tumor volume and tumor weight were measured (**C**). Data were presented as mean ± SD. *n* = 5. **D** Representative IHC staining for CD8 and GZMB in subcutaneous tumors of each group (left). Scale bars: 50 μm. CD8 and GZMB level are quantified (right). *n* = 5. Data were presented as mean ± SD. **E** Schematic representation of the HOXA10 mediated immune evasion. *p < 0.05, **p < 0.01, ***p < 0.001, ****p < 0.0001, ns: not significant

Since HOXA10 expression influences CD8⁺ T cell infiltration and cytotoxicity within tumors, and CD8⁺ T cells are considering the central role of CD8⁺ T cells in antitumor immunity [49–51], we hypothesized that HOXA10 may affect the response to cancer immunotherapy. To test this, we established subcutaneous tumor models in mice using shHoxa10 and shCTL cells, with or without anti-PD-L1 immune checkpoint blockade (ICB) treatment (Fig. 7A). The results demonstrated that shHoxa10 group exhibited significantly suppressed tumor growth following immunotherapy, with treatment response markedly superior to that observed in the shCTL group (Fig. 7B, C). Immunohistochemical staining further revealed that CD8 and GZMB signals were substantially stronger in ICB-treated shHoxa10 tumors than in similarly treated shCTL tumors (Fig. 7D). In summary, our findings demonstrated that inhibiting HOXA10 expression can substantially enhance the therapeutic efficacy of ICB, which may provide a potential breakthrough strategy for addressing clinical immunotherapy resistance.

## Discussion

In cancer, immune evasion is a well-established hallmark of disease progression, characterized by the accumulation of terminally exhausted CD8⁺ T cells that sustain an immunosuppressive microenvironment and contribute to immunotherapy resistance [52]. In contrast, the molecular events initiating immune evasion during the earliest stages of tumor development remain poorly understood. Previous studies relying on highly mutated and aggressive tumor models fail to recapitulate the nascent phase of immune editing. Our study addresses this gap through a murine lung organoid model with defined oncogenic drivers that simulates initial transformation of lung epithelial cells. Cyclic orthotopic transplantation-mimicking intermittent immune pressure-enabled gradual acquisition of immune-evasive traits, providing a unique window into early tumor-immune dynamics.

Central to this process is HOXA10, which we identify as a critical regulator of immune evasion in early LUAD. Although HOXA10’s functions in promoting tumor progression and EMT are well-established in advanced cancers, including LUAD [53], its involvement in tumor immune evasion, particularly during the early stages, is poorly understood. In our study, we found that HOXA10 operates through canonical NF-κB-CCL2 signaling: by binding the Ikbkb promoter and upregulating IKKβ, it activates a pathway widely recognized for its role in pro-tumorigenic inflammation and immune suppression [54, 55]. Subsequent induction of CCL2, a potent chemoattractant for myeloid-derived suppressor cells and M2-like macrophages [56], directly connects HOXA10 to the recruitment of immunosuppressive myeloid populations. While the role of the NF-κB-CCL2 signaling in recruiting tumor-promoting macrophages is well-established in various cancers [57, 58], our work reveals HOXA10 as a previously unrecognized upstream transcriptional regulator that initiates this pathway during early immune programming. These results corroborate clinical observations linking high CCL2 expression to poor prognosis and immune exclusion in multiple cancers [59, 60].

HOXA10 inhibition markedly remodeled the myeloid compartment, shifting macrophage polarization from an M2-like (e.g., *Chil3, Cx3cr1*) to an M1-like (e.g., *Ccl8, Isg15*) phenotype, while downregulating ECM-remodeling enzymes such as *Mmp13* and *Fn1*. These alterations suggest that HOXA10 orchestrates both immunological and physical barriers, mediated by immunosuppressive macrophages and desmoplastic ECM deposition that collectively exclude cytotoxic lymphocytes [45, 46]. Furthermore, HOXA10-deficient tumors fostered structured macrophage-CD8⁺ T cell niches, characterized by increased spatial co-localization of F4/80⁺ and CD8⁺ cells. Such stromal niches are essential for antigen presentation and sustaining T cell cytotoxicity, which are increasingly recognized as prerequisites for effective immunotherapy [47–50].

The mechanisms for the rapid induction of HOXA10 expression in vivo during early LUAD progression represent an important question for future investigation. Our longitudinal monitoring of AHS-T2 cells revealed a stably elevated expression of HOXA10 over a 35-day in vitro period compared to the parental AP-T1 cells (Figure S10C). This sustained high expression, corroborated by the stable high HOXA10 phenotype in cells recovered from multiple in vivo rounds (Fig. 2D), suggesting its upregulation is an integral part of the early transformation process rather than a stochastic event. Further ATAC-seq analyses revealed increased chromatin accessibility at the HOXA10 promoter in AHS-T2 and AHS-T4 cells compared to AP-T1 (Figure S10D), strongly suggesting epigenetic reprogramming as a key driver, a process increasingly recognized as a pioneer event in tumor evolution [61–63]. This reprogramming is likely triggered by a combination of intrinsic and extrinsic signals from the nascent tumor microenvironment, such as local hypoxia and inflammation, which may recruit pioneer transcription factors or chromatin remodelers to the locus [64, 65]. Future studies are warranted to delineate the precise mechanisms leading to increased chromatin accessibility of HOXA10 during initial tumor development.

The synergistic effect between HOXA10 inhibition and anti-PD-L1 therapy underscores the translational potential of this pathway in overcoming immune resistance. HOXA10 emerges as a master regulator of an immune-evasive program in early LUAD, influencing both tumor-intrinsic signaling and stromal organization. Future work would validate the HOXA10-CCL2 axis in human precancerous and early-stage LUAD lesions, especially in relation to immunotherapy response.

## Conclusions

Our study demonstrated the significant role of HOXA10 in tumor immune evasion during the tumor initiation stage. HOXA10 enhances CCL2 secretion via canonical NF-κB signaling to establish an immunosuppressive tumor microenvironment, thereby significantly augmenting the immune evasion capacity of tumor cells. In summary, this study unveils a key mechanism underpinning immune escape during the initial development of lung adenocarcinoma and underscores the critical need to identify and target sources of immune tolerance in early-stage disease.

## Supplementary Information

Supplementary Material 1Supplementary Material 2Supplementary Material 3

## Data Availability

The data that support the findings of this study are available from the corresponding author upon reasonable request.

## Abbreviations

LUAD: Lung adenocarcinoma
NSCLC: Non-small cell lung cancer
ICB: Immune checkpoint blockade
AAH: Atypical adenomatous hyperplasia
AIS: Adenocarcinoma in situ
MIA: Minimally invasive adenocarcinoma
CTLs: Cytotoxic T cells
LOH: Loss of heterozygosity
CNV: Copy number variation
PCA: Principal component analysis
TGEA: Time-series Gene Expression Analysis
TCGA: The Cancer Genome Atlas
GSEA: Gene Set Enrichment Analysis
TIB: Tumor-infiltrating barrier
BMDMs: Bone marrow-derived macrophages
ELISA: Enzyme-linked immunosorbent assays
ChIP: Chromatin immunoprecipitation
