# Supplementary material for: HOXA10 drives immune evasion in early lung adenocarcinoma by recruiting immunosuppressive macrophages via NF-κB/CCL2 signaling
Source: J Exp Clin Cancer Res. 2026 Feb 25;45:84. doi: 10.1186/s13046-026-03679-6 (PMC13041504; doi:10.1186/s13046-026-03679-6)
Supplement: Supplementary file 1 — Supplementary Material 1Supplementary Material 2Supplementary Material 3 [file 13046_2026_3679_MOESM1_ESM.zip › 13046_2026_3679_MOESM1_ESM/Supplementary Methods and Figures.docx]

**Supplementary Methods**

**Western Blot primary antibodies**: rabbit polyclonal anti-Hoxa10 (1:1,000, Santa Cruz, #sc-271428), rabbit polyclonal anti-p65 (1:1,000, Proteintech, #10745-1-AP), rabbit polyclonal anti-IKKβ (1:1,000, CST, #8943T), rabbit polyclonal anti-CCL2 (1:1,000, Proteintech, 26161-1-AP), and mouse monoclonal anti-GAPDH (1:10,000, Proteintech, #60004-1-Ig).

**Primary antibodies for immunohistochemistry** : anti-CD8a (1:1,000, Abcam, ab217344), anti-PD-L1 (1:1,000, Abcam, ab217344), anti-CD163 (1:400, Proteintech, 83285-4-PBS), anti-GZMB (1:500, R&D, AF1865), anti-FOXP3 (1:1,000, Invitrogen, 14-5773-82), anti-CCL2 (1:400, Proteintech, 26161-1-AP), anti-collagen I (1:1,000, Abcam, ab21286), and anti-F4/80 (1:500, Invitrogen, 14-4801-82).

**Antibodies for FCM**: anti-CD3e (Invitrogen, #11-0031-81), anti-CD8a (Invitrogen, #416-0086-80), anti-F4/80 (BioLegend, #157305), anti-CD86 (BioLegend, #105123), and anti-CD163 (BioLegend, #156703).

**CCK8 assay**: For CCK8 assay, AP and AHS group cells were cultured in 96-well plates (1,000 cells per well) for 1 week. Cell viability was tested at day 0, 1, 3, 5, 7 according to manufacturer’s instructions (Vayzme, #A311-01).

**Plate colony formation assay**: AP and AHS group cells were seeded in 6-well plates (1,000 per well) and cultured for 2 weeks. Then cells were fixed with 4%PFA and stained with 0.2% Crystal violet.

**Soft agar colony formation assay**: AP and AHS cells were seeded at a density of 5,000 cells per well in a 1.5 mL top layer of 0.45% agar. This mixture was layered over a 1.5 mL solidified base layer of 0.7% agar in 6-well plates. Complete culture medium was replenished every 3 days. After approximately 3 weeks, or macroscopic colonies became visible, the colonies were fixed and stained with 0.01% Crystal Violet for visualization and quantification(1, 2).

**ATAC-seq**: 50000 cells were used for ATAC-seq library preparation according to manufacturer’s instructions (Vayzme, #TD711). Paired-end ATAC-seq reads were aligned to the GRCm39 mouse reference genome using Bowtie2 v2.5.4(3). PCR duplicates were identified and removed using GATK MarkDuplicates v4.1.0 under default settings. BAM files were merged using Samtools, and Tn5 insertion offsets were corrected using alignmentSieve from the deepTools v 3.5.3 suite (4). Genome-wide coverage tracks were generated using bamCoverage with RPGC normalization. Peaks were called on merged BAM files using MACS2 v2.2.1(5).

**Somatic variant calling and analysis:** Somatic single nucleotide variants (SNVs) and small insertions/deletions (indels) were identified using GATK Mutect2 (v4.1.0) with base quality score recalibrated BAM files from gene knockout and wild-type samples. For each comparison, the knockout sample at different time points was analyzed against the corresponding wild-type control. Candidate variants were first filtered using GATK FilterMutectCalls (v4.1.0) with default parameters. High-confidence variants were then functionally annotated with ANNOVAR(6). VCF files were converted into ANNOVAR input format using convert2annovar.pl (with --filter pass to retain only variants passing quality filters), followed by annotation with table_annovar.pl against the GRCm39 (mm39) reference database using the refGene protocol(7). Gene-based annotations were generated with default parameters, and missing values were denoted as “.”. Annotated somatic variants were analyzed using the R package maftools (v2.24.0)(8). We quantified the total number of mutations (SNVs and indels) per sample, classified variants by type (e.g., transitions, transversions, insertions, deletions), and identified high-frequency mutated genes (genes with recurrent or high numbers of mutations across samples).

**Copy number analysis:** Copy number alterations were inferred using CNVkit (v0.9.11)(9). Copy number ratios were estimated as log2-transformed values relative to the reference. At the chromosome and gene levels, thresholds of log2 > 0.3 and log2 < −0.4 were applied to define copy number gains (amplifications) and losses (deletions), respectively.

**TCGA LUAD RNA-seq data:** RNA-seq data for lung adenocarcinoma (LUAD) were downloaded from The Cancer Genome Atlas (TCGA) portal using gdc-client(10). The raw count files were aggregated and processed to construct a gene-level expression matrix.

**Early and advanced adenocarcinoma RNA-seq data:** Public RNA-seq datasets of early and advanced adenocarcinomas were obtained from the DBKERO database (download page: https://kero.hgc.jp/Early_cancer.html). The downloaded expression data were curated and converted into a gene-level expression matrix.

**Analysis of RNA-seq data:** Differentially expressed genes (DEGs) were identified with DESeq2 (v1.46.0)(11), using thresholds of adjusted P value < 0.05 and absolute log_2fold change ≥ 1. For visualization and group-wise comparison of specific gene expression levels, variance stabilizing transformation (VST)-normalized values generated by DESeq2 were used, while statistical significance was assessed using DESeq2-derived P values. Functional enrichment analyses of DEGs were performed with clusterProfiler (v4.2.2)(12), including GO (enrichGO)(13) and KEGG (enrichKEGG)(14) pathway analyses. In addition, gene set enrichment analysis (GSEA) was conducted using clusterProfiler with GO and KEGG databases, applying an adjusted P value < 0.05 as the significance cutoff. The Molecular Signatures Database (MSigDB, http://www.gsea-msigdb.org/gsea/msigdb/index.jsp) was used to retrieve pathway-related gene sets, with “HALLMARK_EPITHELIAL_MESENCHYMAL_TRANSITION” selected as the EMT-associated gene set. The EMT score of each sample was then calculated using the single-sample Gene Set Enrichment Analysis (ssGSEA) method implemented in the R package “GSVA” v1.52.3(15).

**Time-series Gene Expression Analysis:** Time-series clustering and visualization of gene expression profiles were performed using ClusterGVis (v0.99.0)(16). As input, the RPKM expression matrix of differentially expressed genes across groups was used. Genes were partitioned into nine distinct expression clusters based on temporal patterns. Subsequently, the genes within each cluster were subjected to Gene Ontology (GO) enrichment analysis to characterize the biological processes associated with distinct temporal expression trajectories.

**scRNA-seq Library Preparation:** Fresh mouse tumor tissues were cut into approximately 1 mm³ pieces in RPMI-1640 medium (Gibco) supplemented with 10% FBS (Gibco), and enzymatically digested using the Tumor Dissociation Kit (Miltenyi, 130-095-929) for 60 minutes on a rotor at 37°C. Dissociated cells were subsequently passed through 100-μm MACS SmartStrainers (Miltenyi, 130-110-917) and centrifuged at 300g for 8 minutes. After removing the supernatant, pelleted cells were resuspended in red blood cell lysis buffer (Sangon) and incubated on ice for 5 minutes to lyse red blood cells. After washing with PBS (Gibco), cell pellets were resuspended in sorting buffer (PBS supplemented with 2% FBS). Single-cell suspensions were stained with 0.4% Trypan Blue solution (Gibco) for viability assessment. CD45⁺ cells were enriched by positive selection using CD45 MicroBeads (Miltenyi, 130-052-301). The concentration of single-cell suspensions was adjusted to 700-1,200 cells/ml, and single-cell suspensions with viable cell rates greater than 80% were used for single-cell sequencing. A total of 20,000 cells per sample were used for 10× Chromium single-cell 5' and mouse TCR library construction (10× Genomics) according to the manufacturer's instructions. All subsequent steps were performed following standard manufacturer protocols. Purified libraries were sequenced using an Illumina NovaSeq 6000 sequencer with 150-base pair (bp) paired-end reads.

**Supplementary Figures:**

**Figure S1. Genotyping and cell culture of early-stage LUAD cells.** (A) Genotyping by PCR analysis of Cre-mediated recombination. (B) Representative merge, EGFP and bright field channel for 2D-Cultured EGFP+ Cells. Scale bars: 200 μm. (C) Schematic representation of the “in vivo cycle” system. Representative merged EGFP images of recovered cells are shown at indicated time points (Day 0, 5, 10, 15, 22, and 30) throughout the enrichment process. Scale bars: 30 μm. (D) Representative merge, EGFP and bright field channel for AHS-T2 and AP-T2 Cell. Scale bars: 100 μm. (E) Representative merge, EGFP and bright field channel for AHS-T3 and AHS-T4 Cell. Scale bars: 200 μm.

**Figure S2. Cell growth properties of AP and AHS group cells.** (A) CCK8 assays of AP group and AHS group cells. Two-way RM ANOVA tests were used. Data at day 0, 1, 3, 5, 7 were presented. n=3 each. (B) CCK8 assays of T2, T3, and T4 cells of AP and AHS group. Two-way RM ANOVA tests were used. Data at day 0, 1, 3, 5, 7 were presented. n=3 each. (C-D) Representative plate colony formation assays of AP and AHS group cells. Colonies were quantified with three replicates (D). (E-F) Representative soft agar colony formation assay of AP and AHS group cells. Colonies of AP and AHS group cells were quantified with three replicates (F) . Data were all presented as mean ±SD. *p < 0.05, **p < 0.01, ***p < 0.001, ****p < 0.0001, ns: not significant.

**Figure S3. WES analysis results.** (A-B) Copy number profiles of AP group (A) and AHS group(B). (C) Copy number quantification for AHS group amplified and deleted genes. (D) Copy number quantification for AP group amplified and deleted genes. (E) Heatmap of clinically relevant LUAD genes copy number. (F) Statistics of mutated genes and variants.

**Figure S4. TGEA analysis of AHS and AP groups.** (A) PCA plot of muti-time point RNAseq data of AP and AHS group. (B) TGEA analysis of AHS group. (C) TGEA analysis of AP group. (D) Schematic of clinical categories for early-stage LUAD human samples (hsa group). (E) TGEA analysis of hsa group. (F) Overall survival analysis of eight additional candidate genes using the TCGA-LUAD cohort. Patients were stratified into high and low expression groups based on the median mRNA expression of each gene. (G) Normalized Hoxa10 expression level of AP group RNAseq data. Data were presented as mean ±SD. Each group n=3. ns: not significant.

**Figure S5. EMT signature of AP and AHS group cells.** (A) EMT score of AP and AHS group cells were quantified using RNA-Seq data. n=3 each. (B-D) Normalized transcriptomic expression level of Chd2 (B), Chd1 (C), and Vim (D). n=3 each. (E) Western Blot analysis of N-Cadherin (N-Cad), E-Cadherin (E-Cad), and Vimentin (VIM) in AP and AHS group cells. Quantification of N-Cadherin and E-Cadherin were presented (right). n=3 each. Data were all presented as mean ±SD. *p < 0.05, **p < 0.01, ***p < 0.001, ****p < 0.0001, ns: not significant.

**Figure S6. Orthotopic pulmonary tumors of shCTL and shHoxa10 group.** (A-B) The orthotopic pulmonary tumors of shCTL and shHoxa10 group (A). Lung to body weight ratio and nodule number per lung (B) were measured. n=5 each. Data were presented as mean ±SD. *p < 0.05, **p < 0.01, ***p < 0.001, ****p < 0.0001, ns: not significant.

**Figure S7. Transcriptomics analysis of shHoxa10 and shCTL groups.** (A) Volcano plot for DEGs of shHoxa10 vs shCTL RNAseq data. (B) UMAP (Uniform manifold approximation and projection) plot for major cell types. (C) Frequency of each major cell types are presented. (D) Dotplot for gene features of macrophages cell subtypes. (E) Mmp19 and Fn1 expression level of each macrophages cell subtypes. Data were presented as mean ±SD. (F) Dotplot for gene features of CD8+ T cell subtypes. (G) Prf1 expression level of each CD8+ T cell subtypes. Data were presented as mean ±SD. (H) Dotplot for gene features of CD4+ T cell subtypes. (I) Frequency of each cell subtypes of CD4+ T cells are presented. (J) All significant ligand-receptor pairs of Cellchat analysis. *p < 0.05, **p < 0.01, ***p < 0.001, ****p < 0.0001, ns: not significant.

**
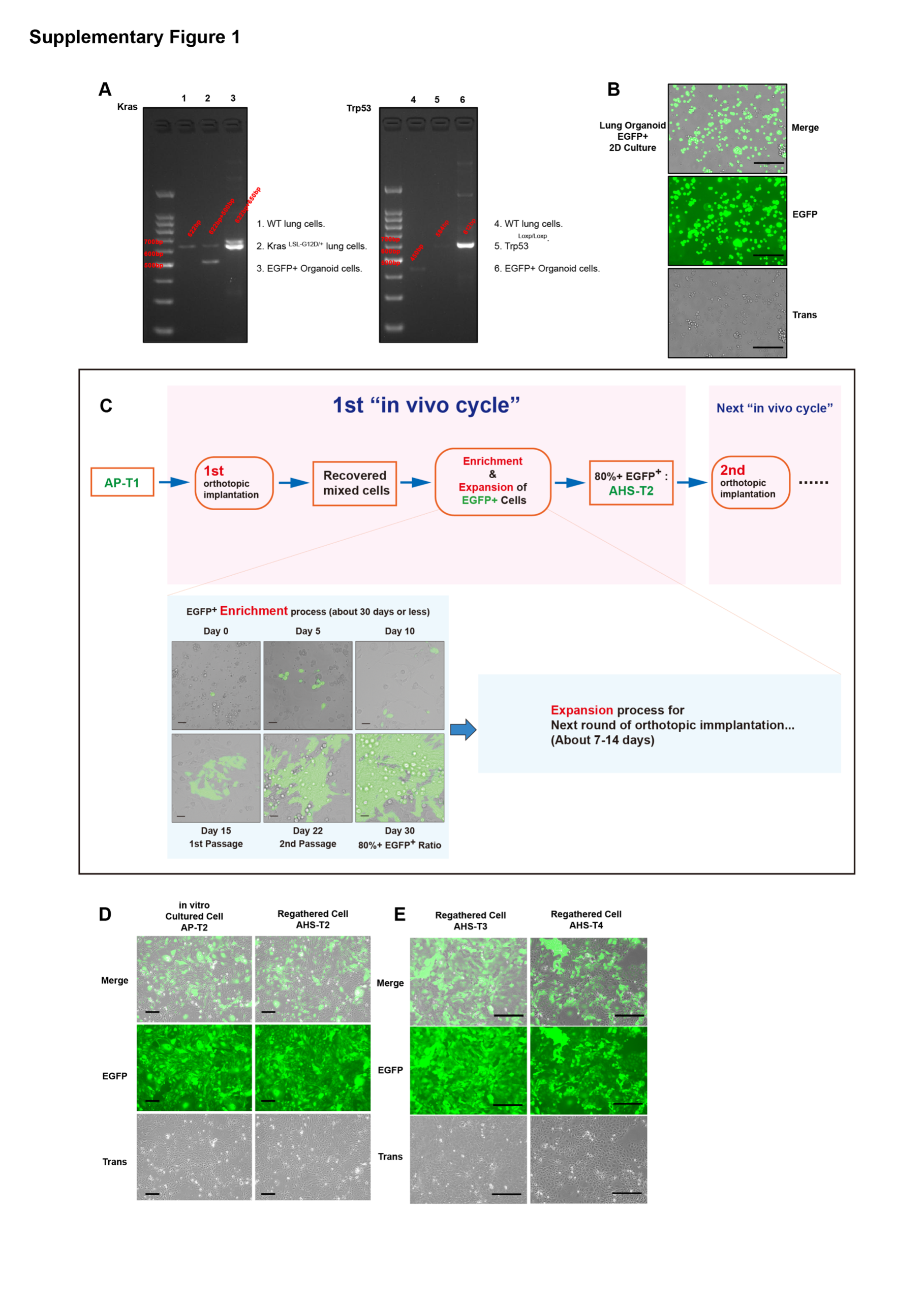
**

**
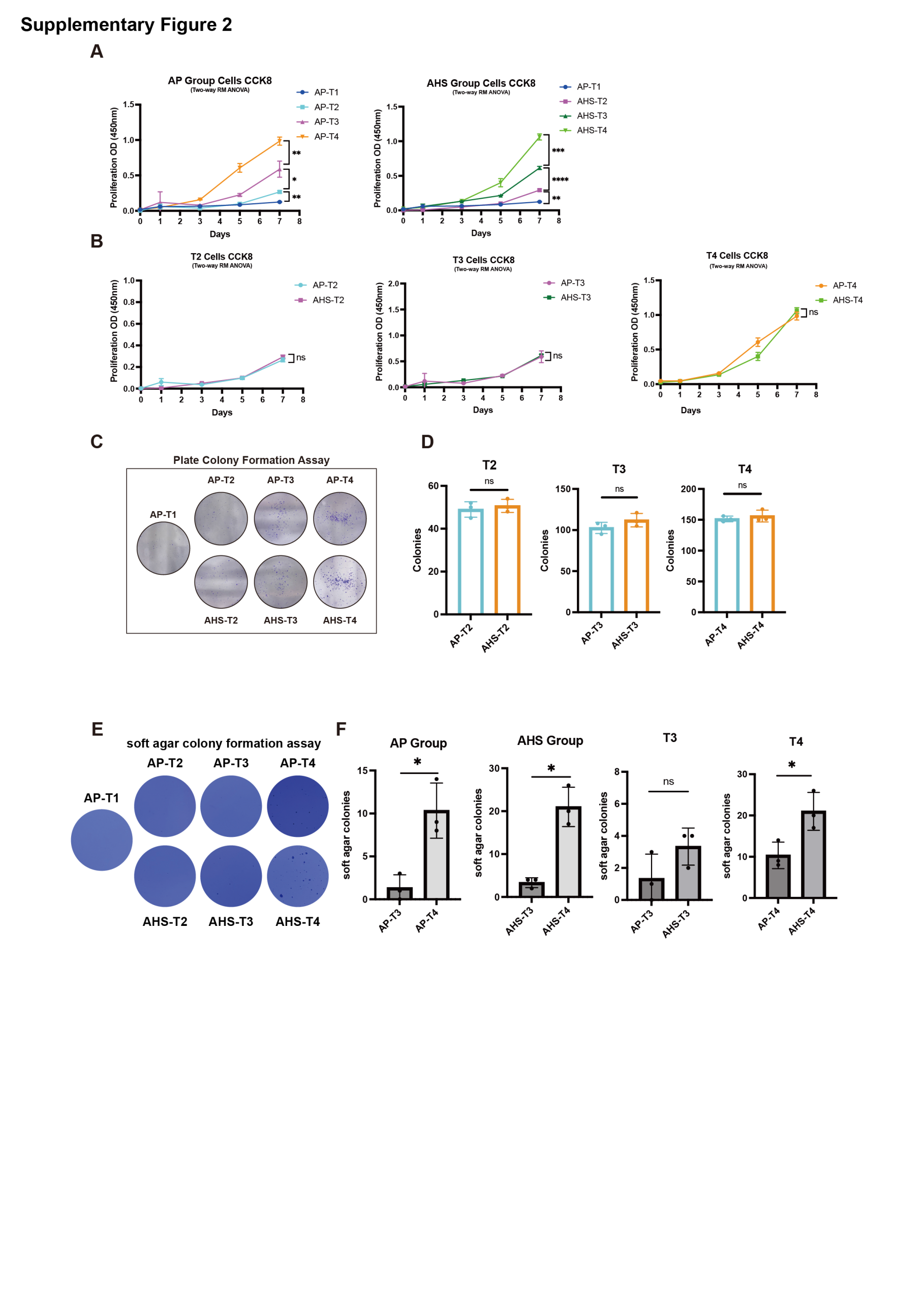
**

**
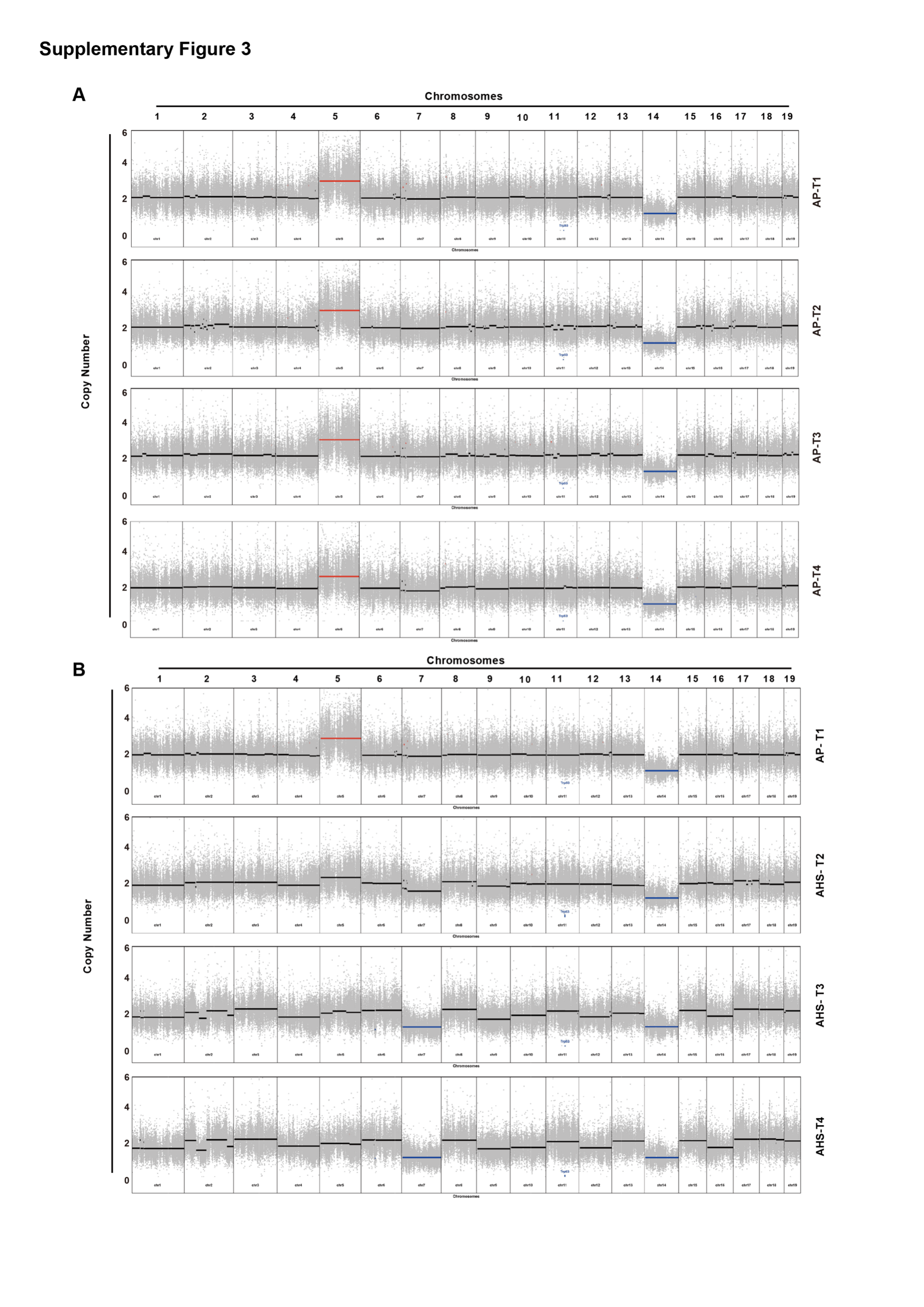
**

**
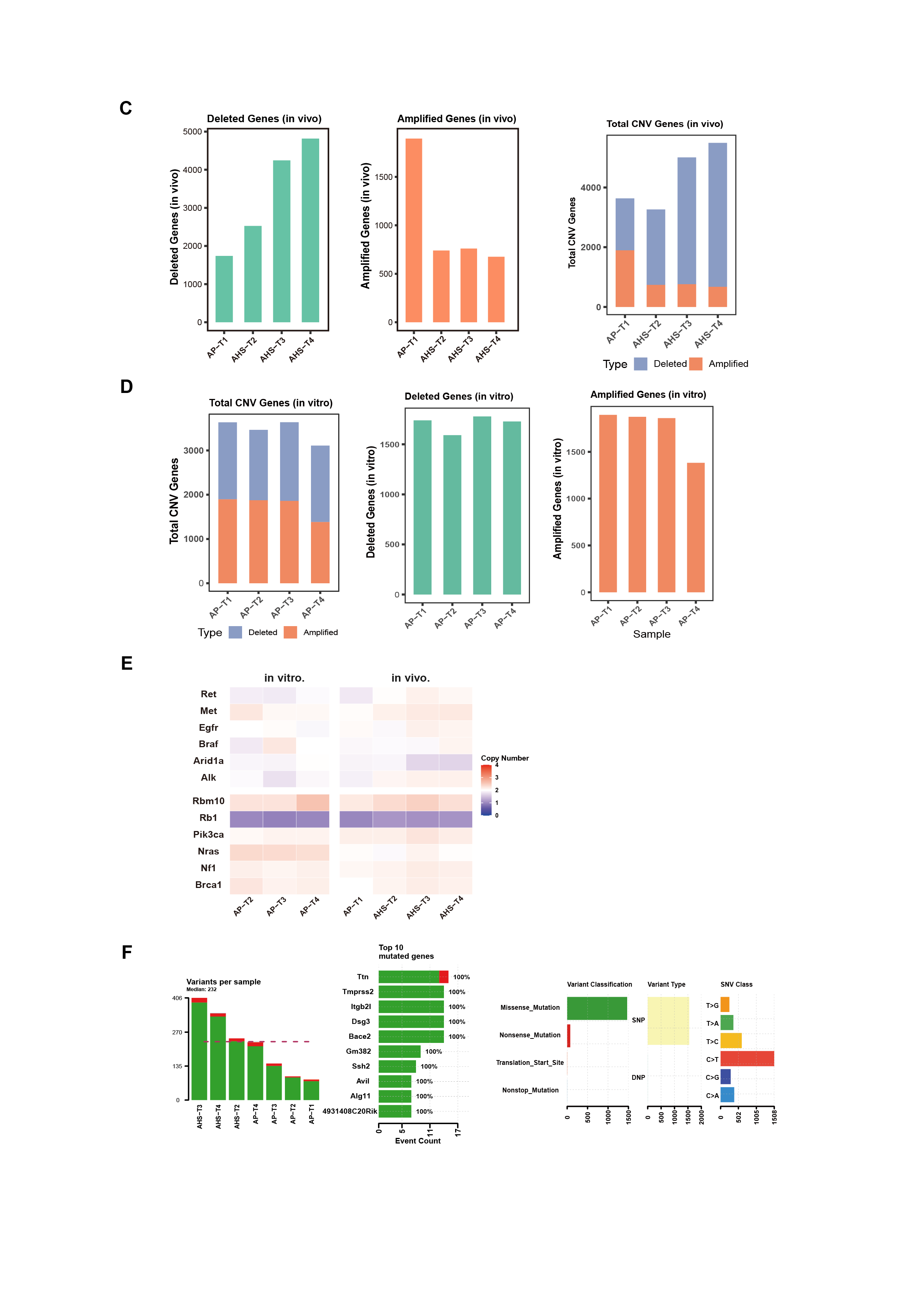
**

**
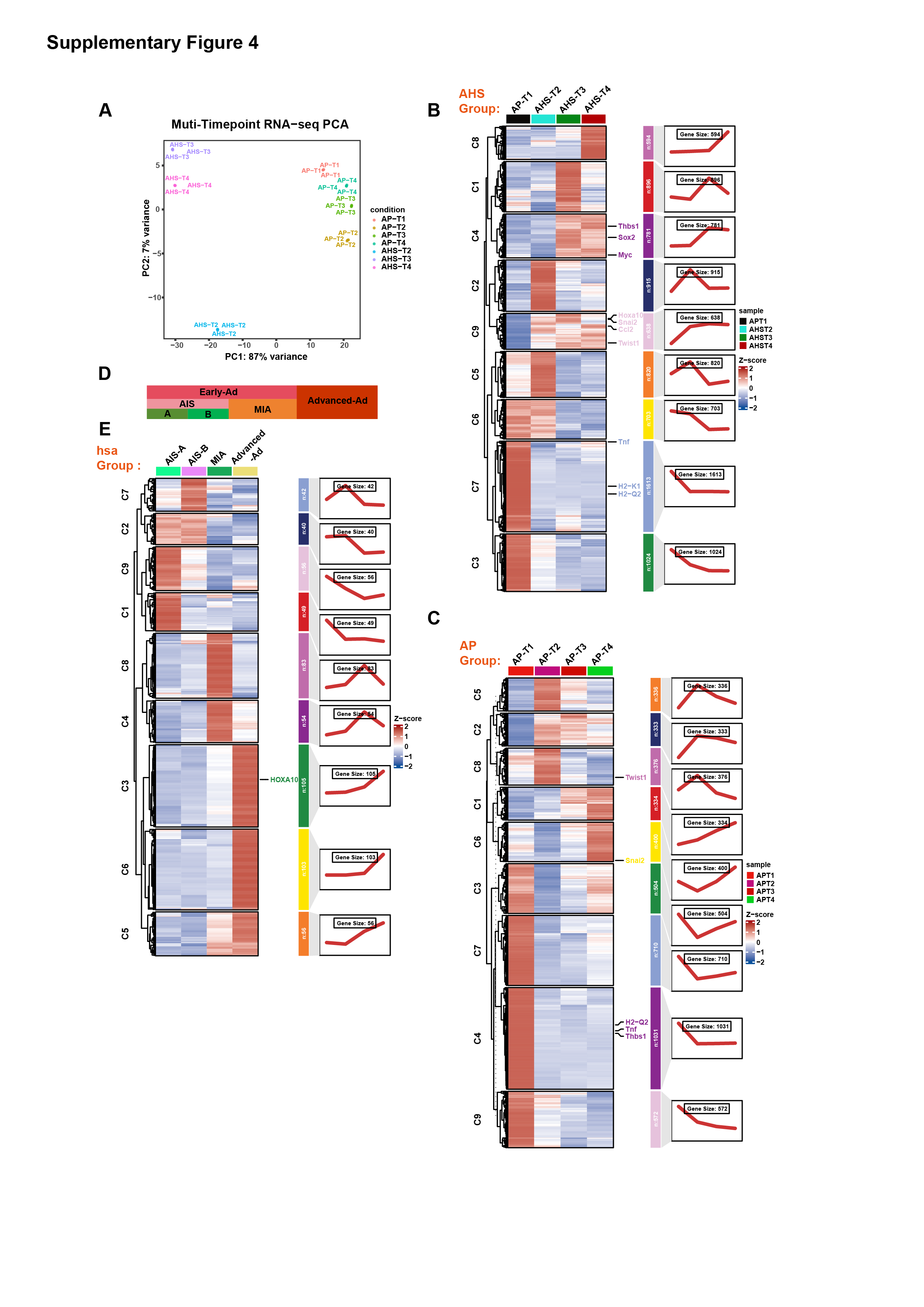
**

**
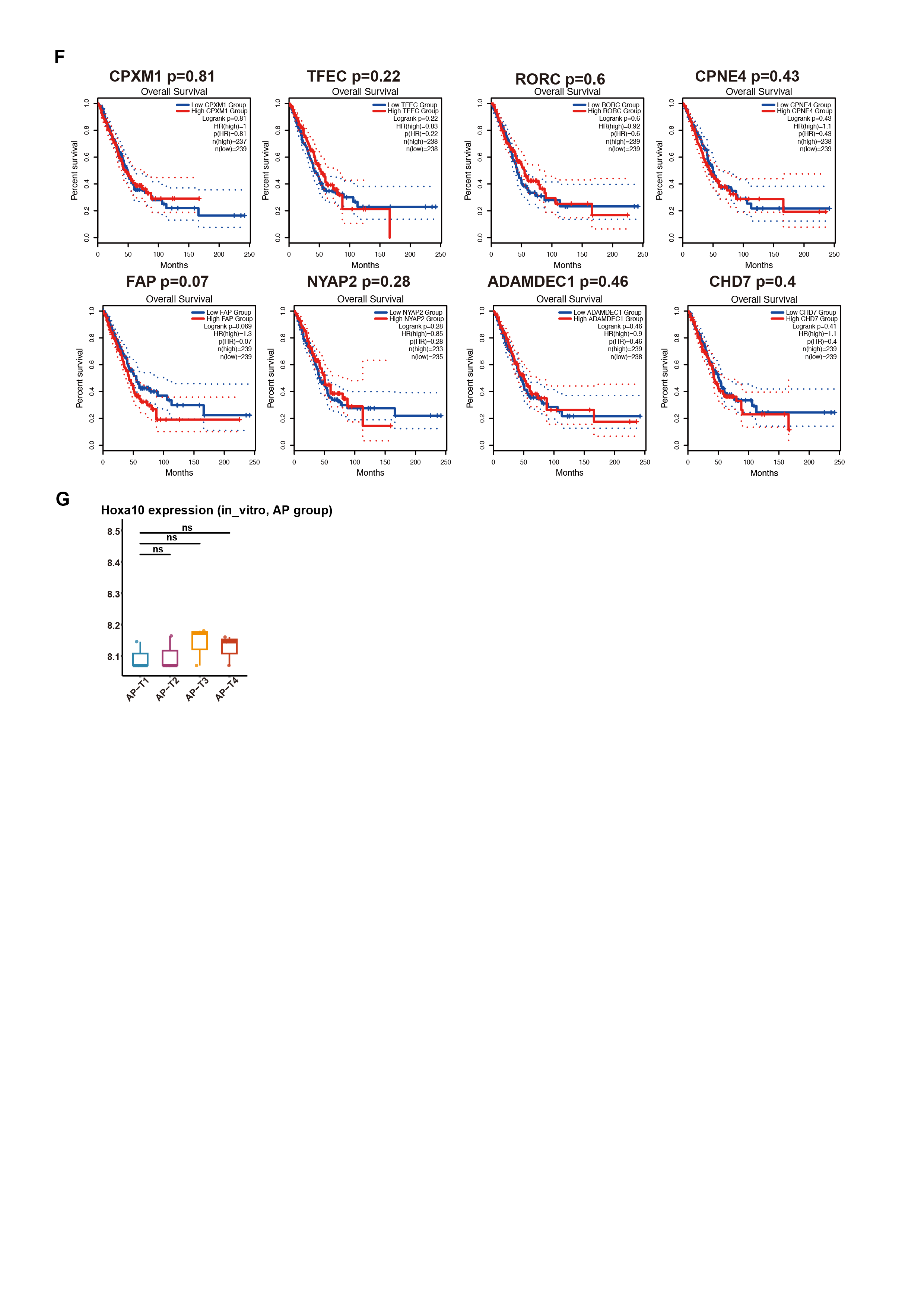
**

**
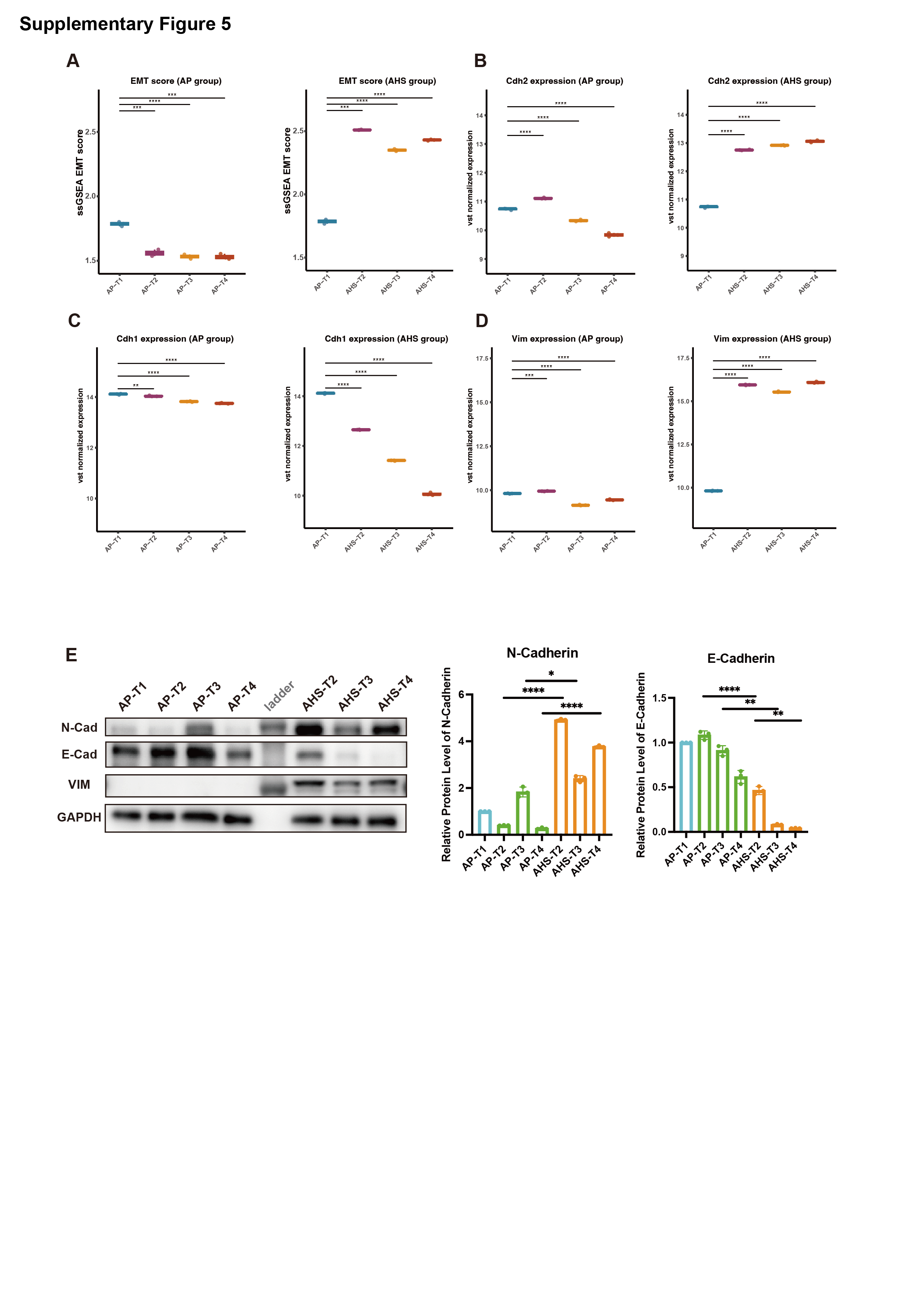
**

**
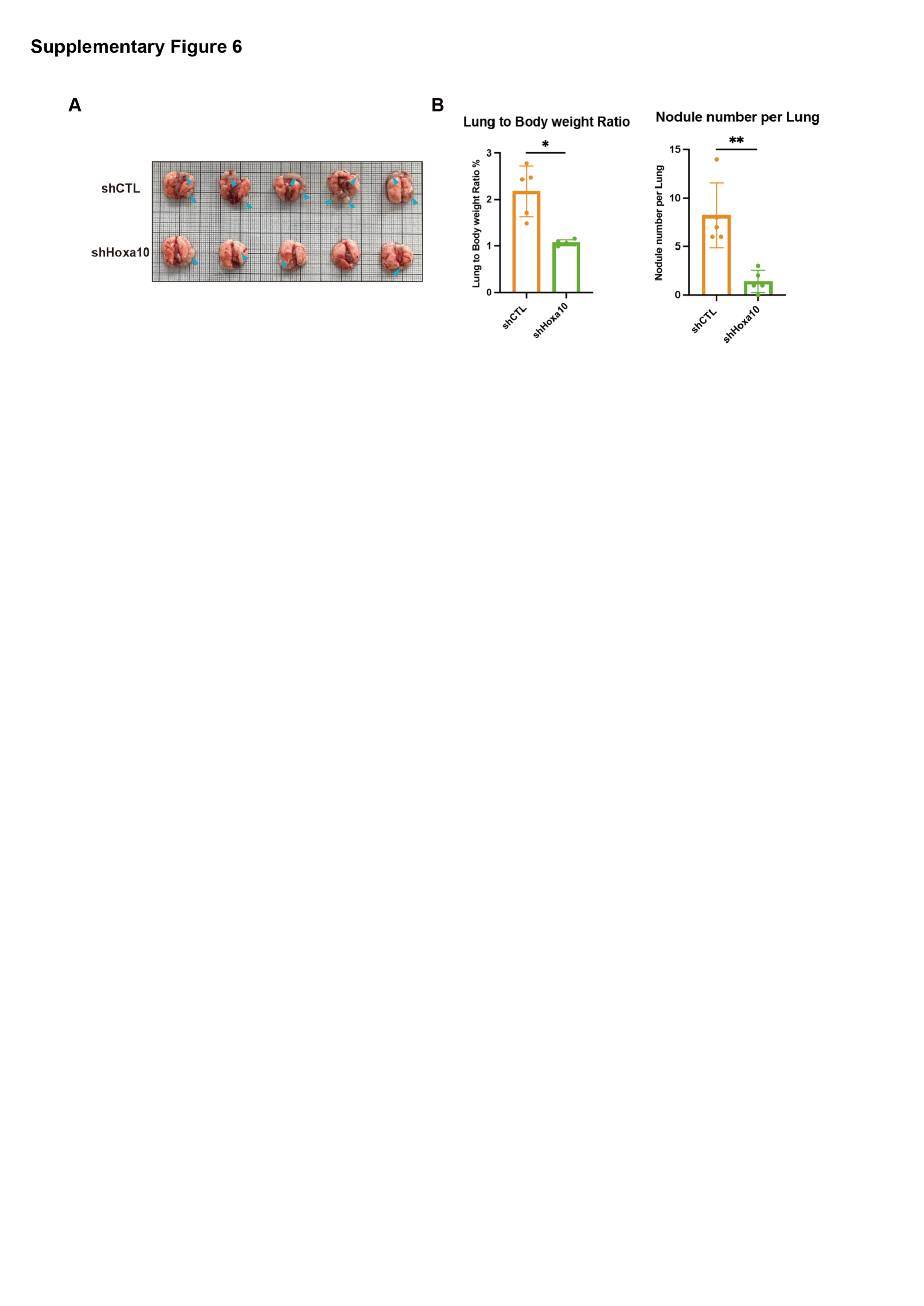
**

**
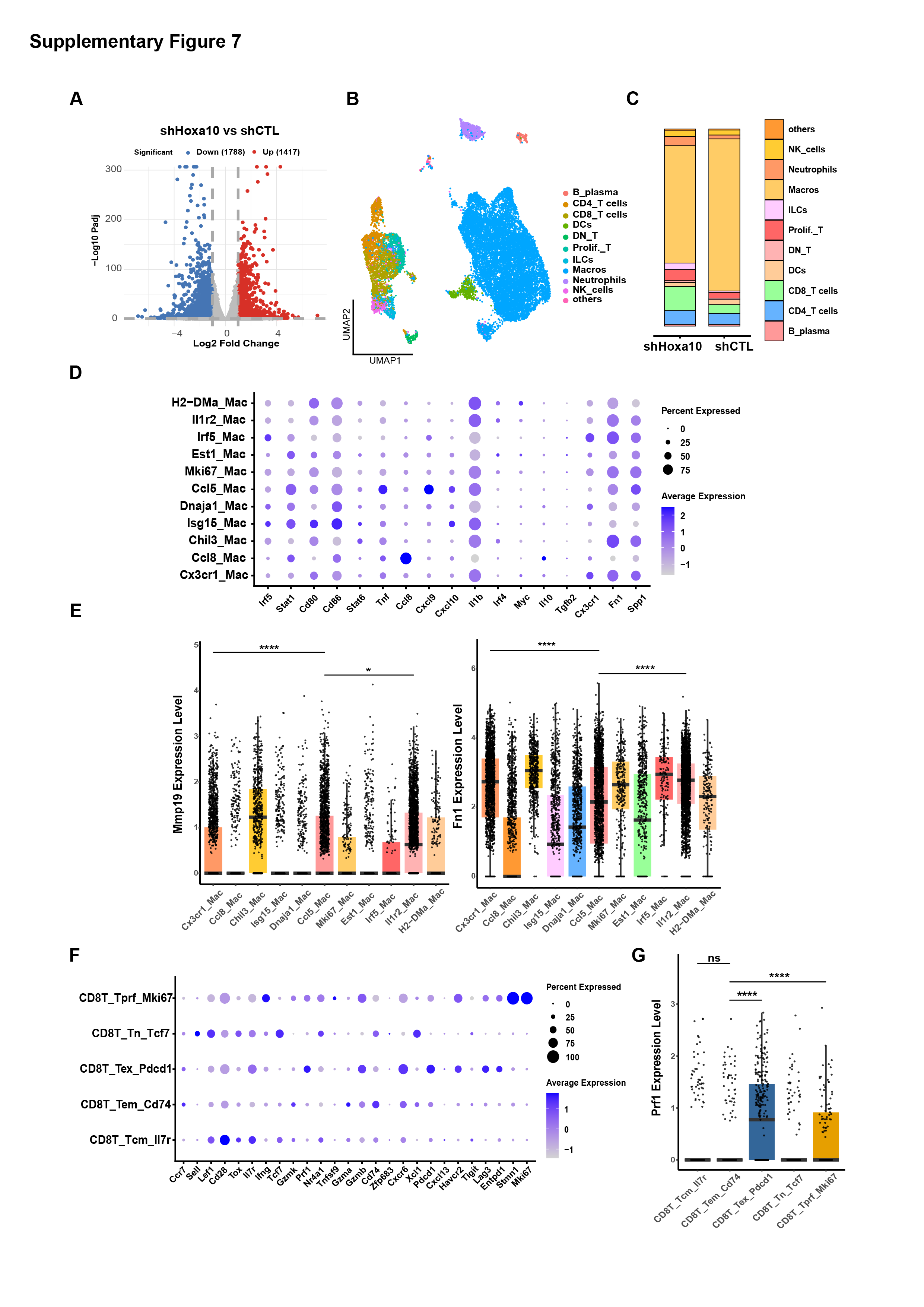
**

**
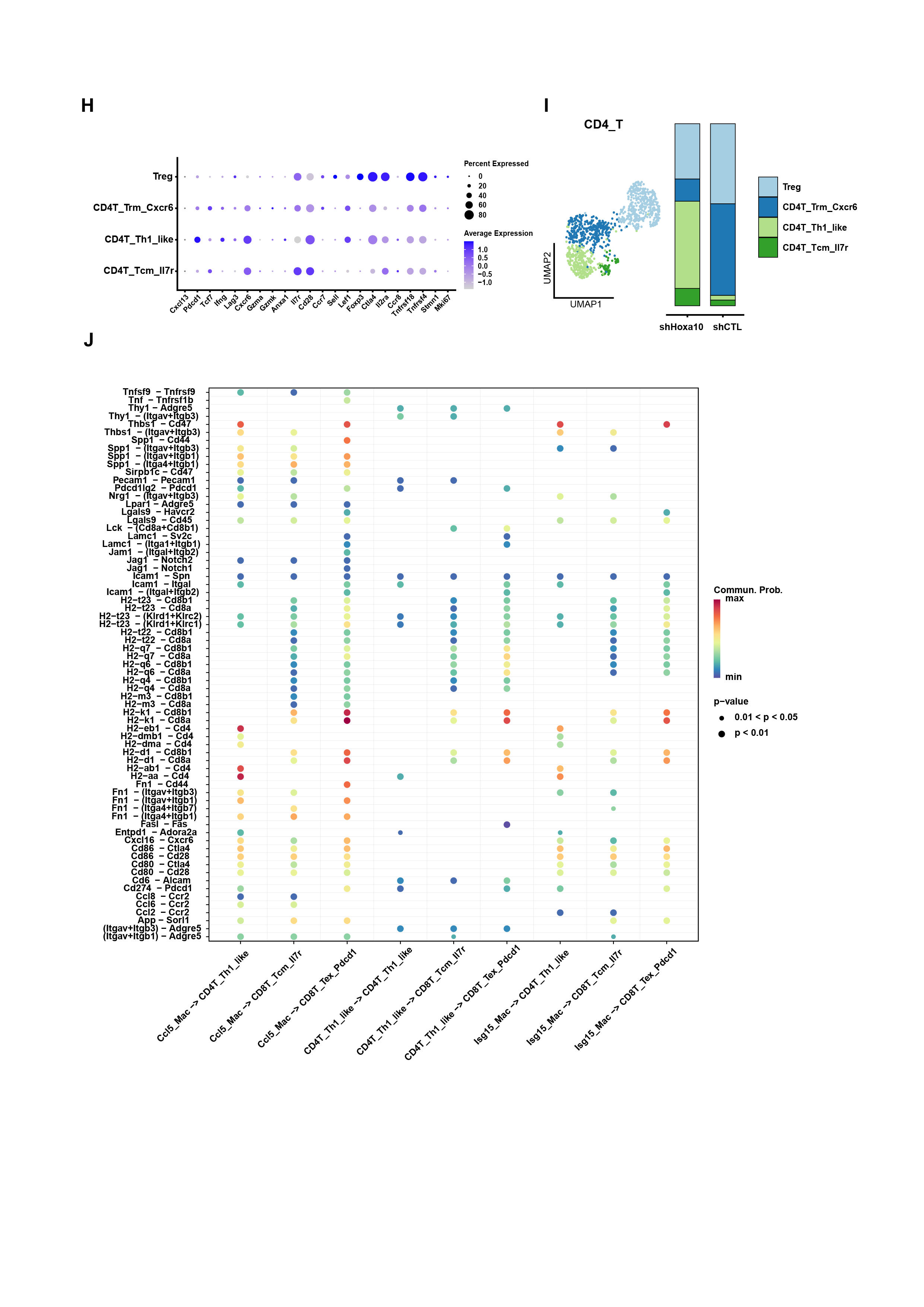
**

**
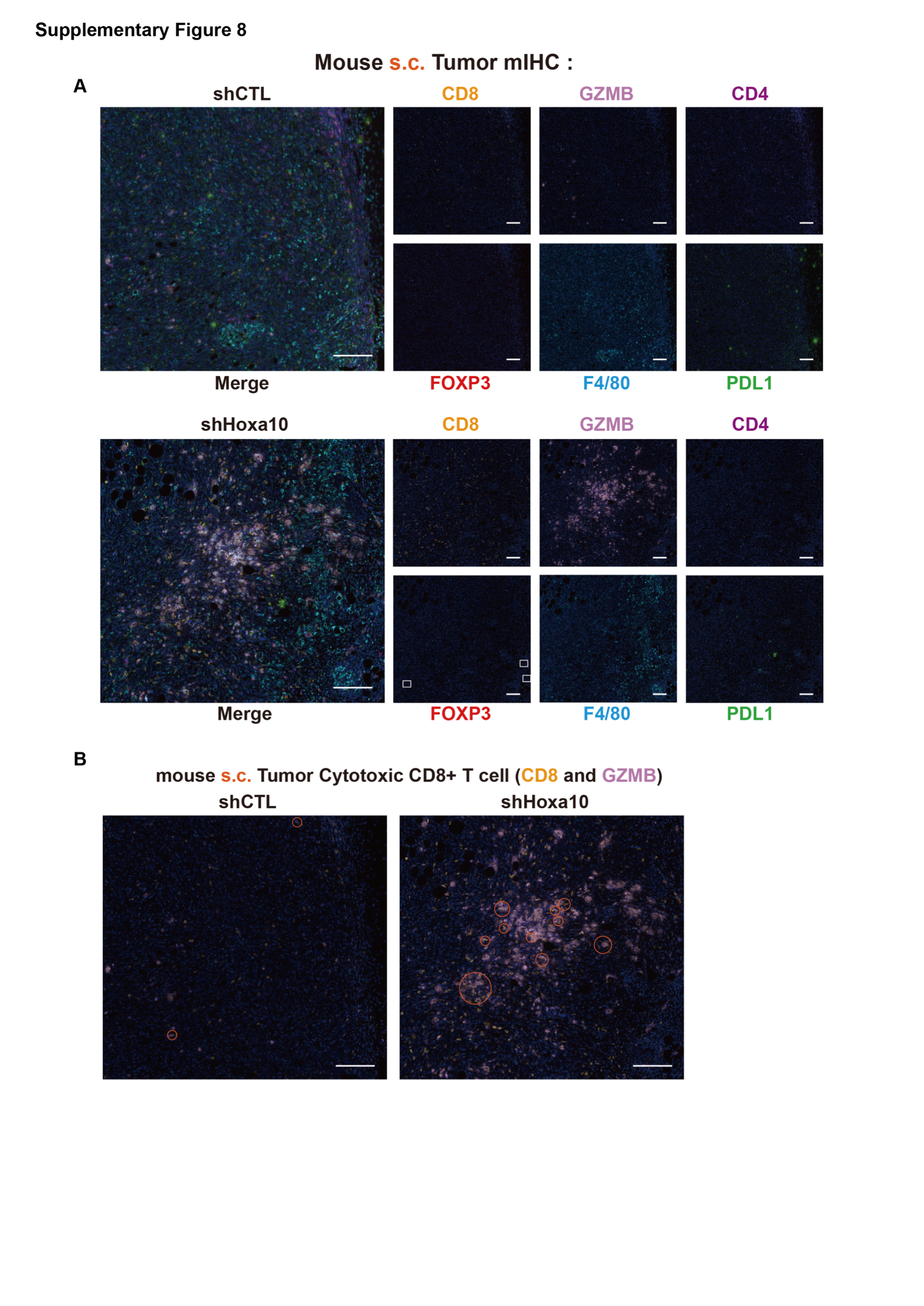
**

**
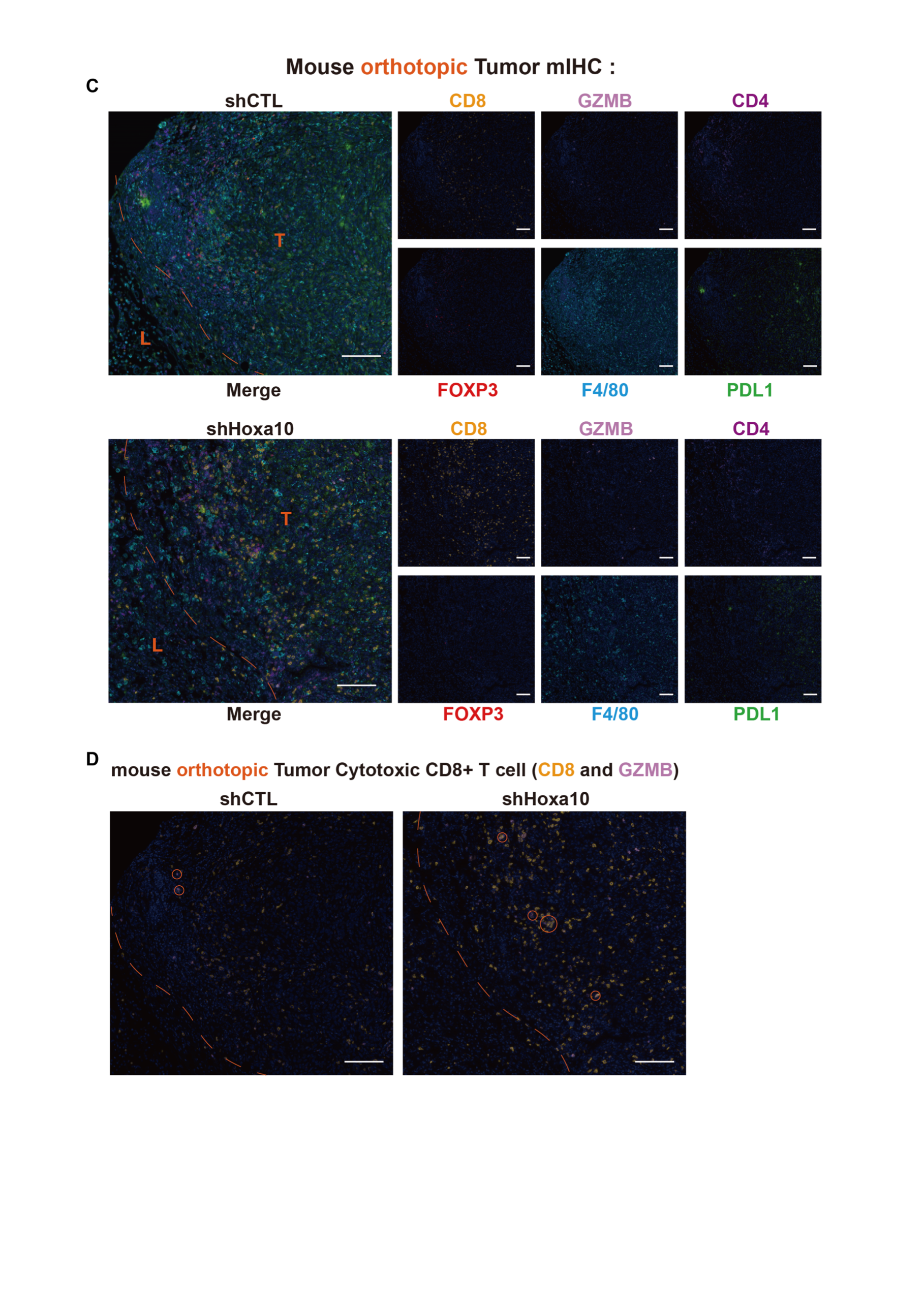
**

**
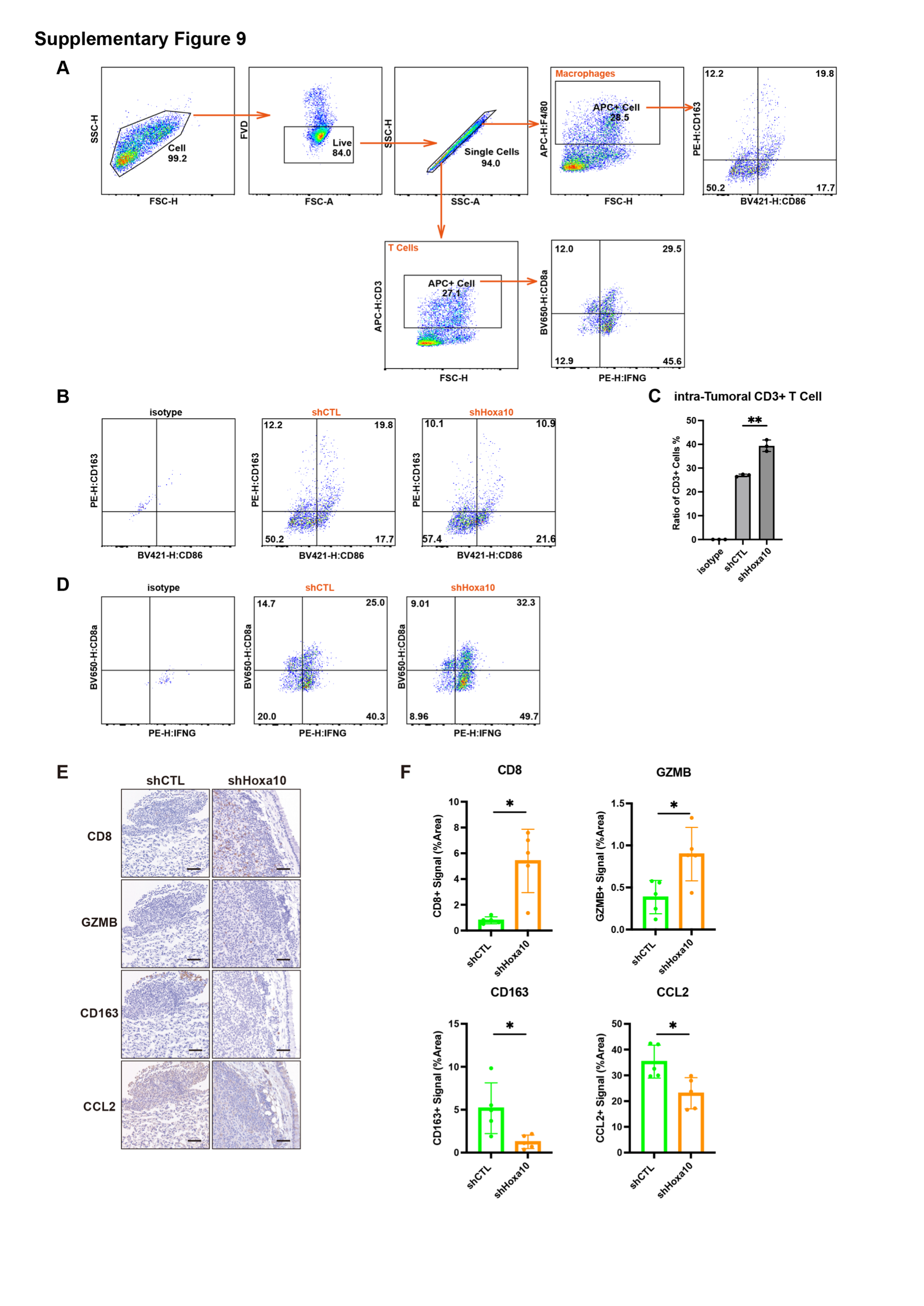
**

**
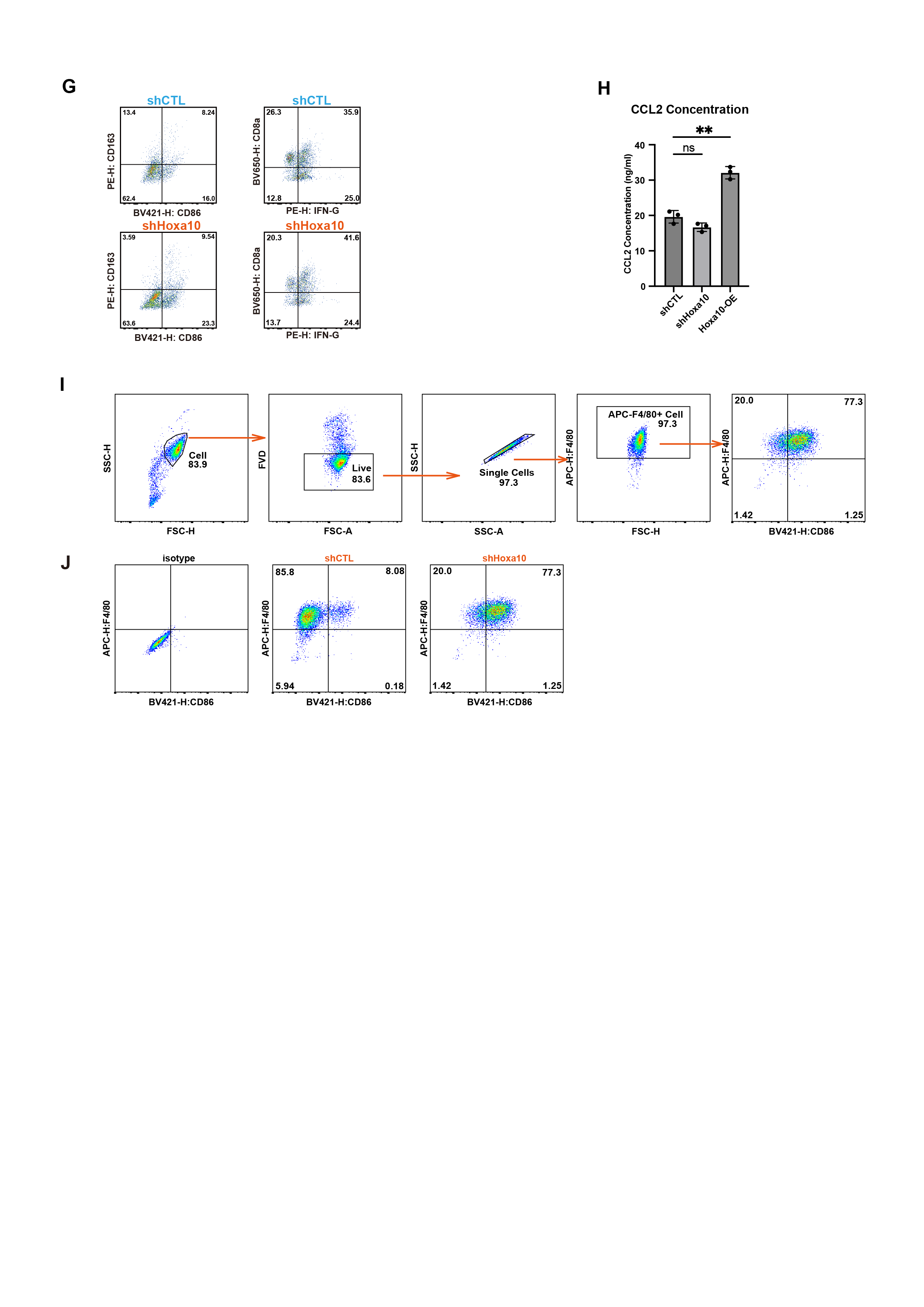
**

**
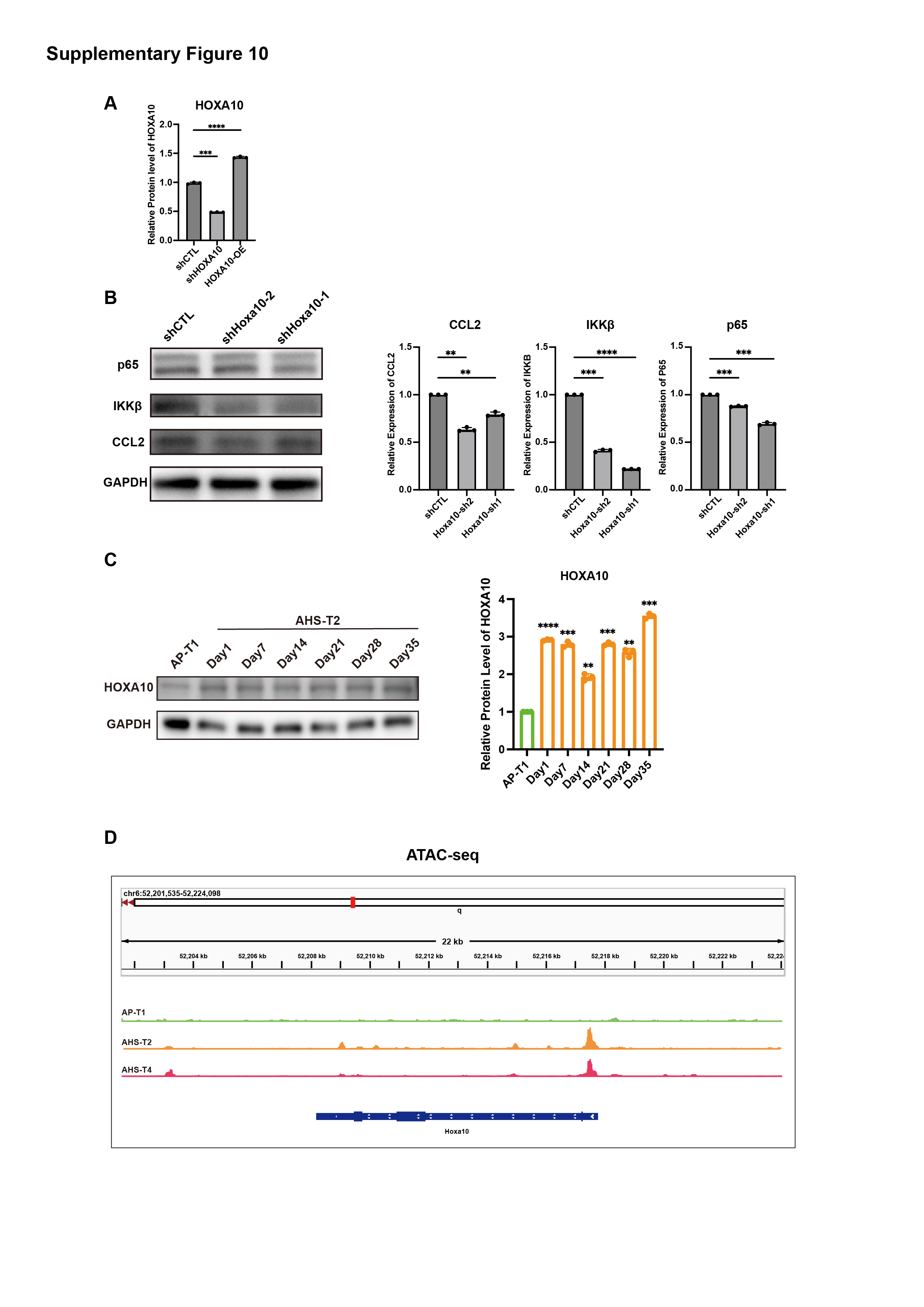
**

**Reference**

1. Stévenin V, Neefjes J. Soft agar colony formation assay to quantify mouse embryonic fibroblast transformation after Salmonella infection. STAR protocols. 09/15/2023;4(3).

2. Stanley Borowicz, Michelle Van Scoyk, Sreedevi Avasarala, Manoj Kumar Karuppusamy Rathinam, Jordi Tauler, Rama Kamesh Bikkavilli, et al. The Soft Agar Colony Formation Assay. Journal of Visualized Experiments : JoVE. 2014 Oct 27(92).

3. Langmead B, Salzberg SL. Fast gapped-read alignment with Bowtie 2 Nature methods. 03/04/2012;9(4).

4. Ramírez F, Ryan DP, Grüning B, Bhardwaj V, Kilpert F, Richter AS, et al. deepTools2: a next generation web server for deep-sequencing data analysis. Nucleic acids research. 07/08/2016;44(W1).

5. Feng J, Liu T, Qin B, Zhang Y, Liu XS. Identifying ChIP-seq enrichment using MACS. Nature protocols. 2012 Sep;7(9).

6. Kai Wang, Mingyao Li, Hakon Hakonarson. ANNOVAR: functional annotation of genetic variants from high-throughput sequencing data. Nucleic Acids Research. 2010 Jul 3;38(16).

7. Tamara Goldfarb, Vamsi K Kodali, Shashikant Pujar, Vyacheslav Brover, Barbara Robbertse, Catherine M Farrell, et al. NCBI RefSeq: reference sequence standards through 25 years of curation and annotation. Nucleic Acids Research. 2025/01/06;53(D1).

8. Anand Mayakonda, De-Chen Lin, Yassen Assenov, Christoph Plass, H Phillip Koeffler. Maftools: efficient and comprehensive analysis of somatic variants in cancer. Genome Research. 2018 Nov;28(11).

9. Talevich E, Shain AH, Botton T, Bastian BC. CNVkit: Genome-Wide Copy Number Detection and Visualization from Targeted DNA Sequencing - PubMed. PLoS computational biology. 04/21/2016;12(4).

10. Allison P. Heath, Vincent Ferretti, Stuti Agrawal, Maksim An, James C. Angelakos, Renuka Arya, et al. The NCI Genomic Data Commons. Nature Genetics 2021 53:3. 2021–02–22;53(3).

11. Michael I Love, Wolfgang Huber, Simon Anders, Michael I Love, Wolfgang Huber, Simon Anders. Moderated estimation of fold change and dispersion for RNA-seq data with DESeq2. Genome Biology 2014 15:12. 2014–12–05;15(12).

12. Guangchuang Yu, Li-Gen Wang, Yanyan Han, Qing-Yu He. clusterProfiler: an R Package for Comparing Biological Themes Among Gene Clusters. OMICS : a Journal of Integrative Biology. 2012 May;16(5).

13. Michael Ashburner, Catherine A. Ball, Judith A. Blake, David Botstein, Heather Butler, J. Michael Cherry, et al. Gene Ontology: tool for the unification of biology. Nature Genetics 2000 25:1. 2000/05;25(1).

14. Minoru Kanehisa, Susumu Goto. KEGG: Kyoto Encyclopedia of Genes and Genomes. Nucleic Acids Research. 2000 Jan 1;28(1).

15. Ferreira MR, Santos GA, Biagi CA, Silva Junior WA, Zambuzzi WF. GSVA score reveals molecular signatures from transcriptomes for biomaterials comparison Journal of biomedical materials research Part A. 2021 Jun;109(6).

16. Jun Zhang. ClusterGVis: One-step to Cluster and Visualize Gene Expression Matrix. 2022 [Available from: <https://github.com/junjunlab/ClusterGVis>.
